# Scalable production of graphene with tunable edge functionalities *via* shear-driven ball milling for energy storage applications

**DOI:** 10.1039/d6na00218h

**Published:** 2026-08-11

**Authors:** Sumisha Surendran, Binitha N. Narayanan

**Affiliations:** a Department of Chemistry, University of Calicut, Calicut University (PO) Malappuram (DT) Thenhipalam Kerala 673635 India binitha@uoc.ac.in +91 494-2407414; b Inter University Centre for Hydrogen & Energy Storage, University of Calicut, Calicut University (PO) Malappuram (DT) Thenhipalam Kerala 673635 India

## Abstract

The scalable production of graphene with controlled chemical functionalities remains a central challenge in translating laboratory advances into practical technologies. Beyond conventional approaches that prioritize the surface area, functionalization or conductivity, increasing attention is being directed toward spatially selective defect engineering that preserves the aromatic π-conjugated carbon framework while enabling targeted interfacial reactivity, thereby providing high-quality graphene. In this context, shear-driven ball milling has emerged as a promising mechanochemical route for the synthesis of edge-functionalized graphene through preferential edge activation and controlled exfoliation. Unlike oxidation-intensive methods that often introduce extensive basal-plane damage, shear-assisted milling promotes layer delamination while largely preserving the intrinsic sp^2^ carbon network. Simultaneously, the mechanochemical environment activates newly generated edge sites, enabling direct reactions with selected milling agents and facilitating controlled incorporation of heteroatoms and functional groups. Such edge-focused functionalization provides an effective means of balancing electrical conductivity, wettability, ion accessibility, and electrochemical activity. This review critically examines the mechanistic principles governing graphite exfoliation during ball milling, the roles of milling agents and processing parameters in regulating structural evolution and surface chemistry, and the characterization strategies used to distinguish edge functionalization from basal-plane modification. Particular emphasis is placed on understanding the relationships between processing conditions, defect generation, functionalization pathways, and electrochemical performance. The influence of edge-engineered graphene on charge storage mechanisms in supercapacitors, lithium-ion batteries, sodium-ion batteries, zinc-ion systems, and hybrid energy-storage devices is comprehensively discussed. In addition, key considerations related to scalability, process economics, sustainability, contamination control, energy consumption, reproducibility, and industrial implementation are evaluated. Overall, this review establishes a process–structure–electrochemistry framework for shear-driven ball-milled graphene and highlights its potential as a scalable platform for the development of advanced graphene materials tailored for next-generation energy-storage technologies.

## Abstract 2

Shear-driven ball milling for scalable production of graphene with controlled chemical functionalities for energy storage applications.

## Graphical abstract

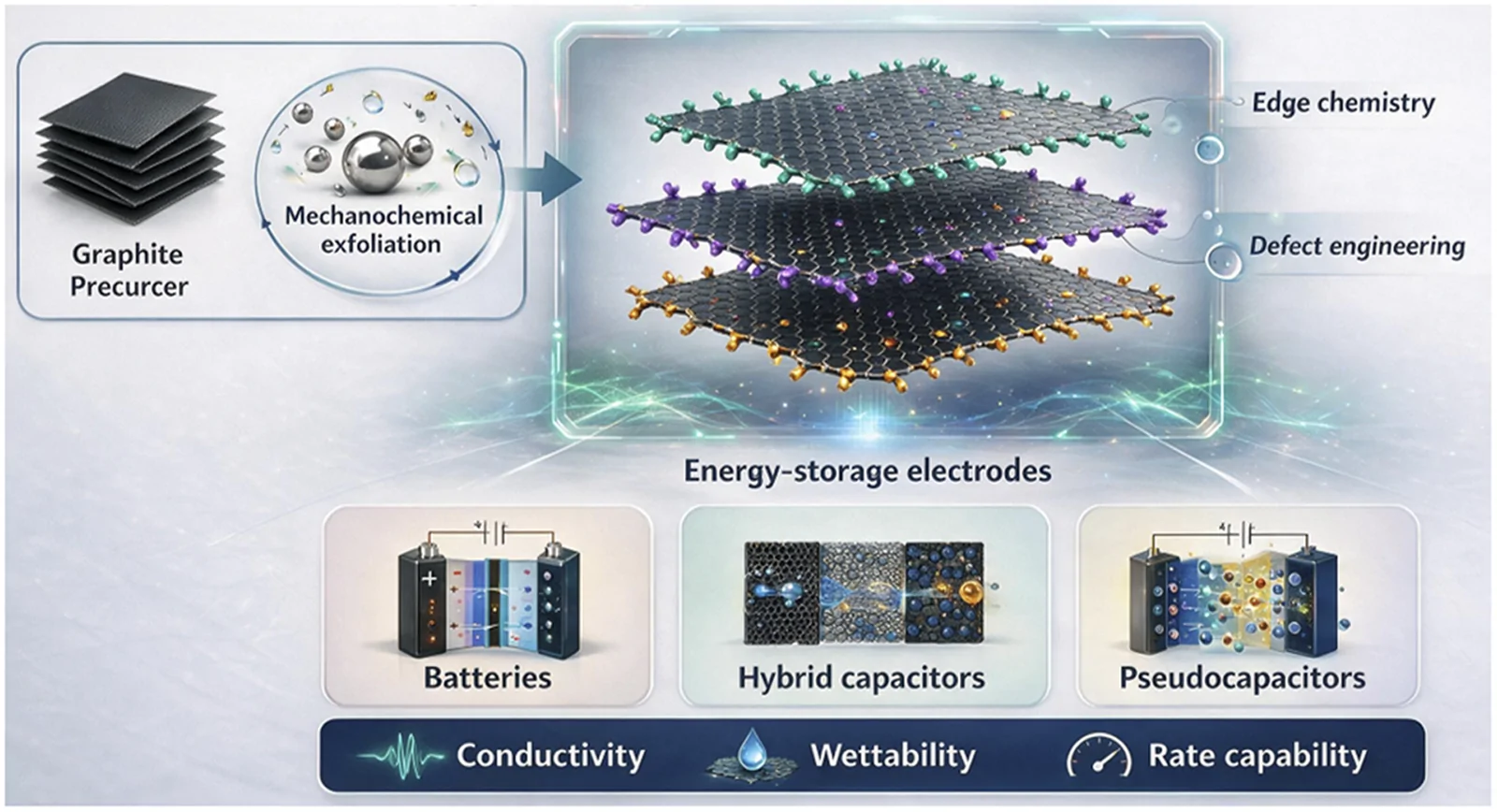

## 1. Introduction: graphene relevance and synthetic strategies

Among low-dimensional materials (LDMs), two-dimensional (2D) sheets (Fig. 1) have attracted particular attention due to their high surface area, unique electronic/optical properties, mechanical strength, *etc.*^1^ Graphene, a single layer of sp^2^-hybridized carbon atoms arranged in a honeycomb lattice, represents the most prominent example of this class.^2^

**Fig. 1:**
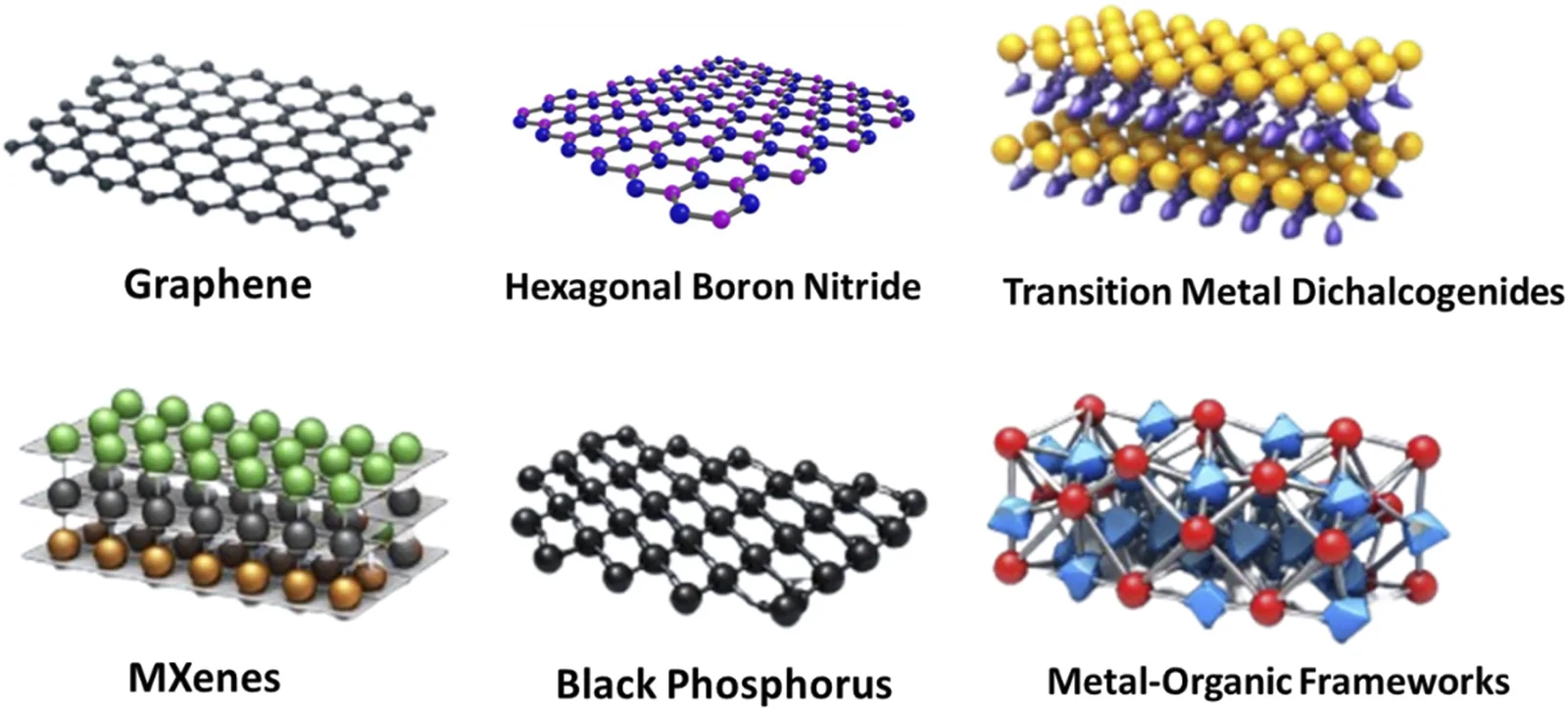
Representation of different types of 2D materials.

Graphene also serves as the fundamental building block for other carbon allotropes, including zero-dimensional fullerenes and graphene quantum dots, one-dimensional carbon nanotubes, and three-dimensional graphite.^3^ Owing to its exceptional electrical conductivity (∼10^3–8^ S m^−1^), high mechanical strength (∼130 GPa), superior thermal conductivity (∼4000 W m^−1^ K^−1^), and large theoretical surface area (∼2630 m^2^ g^−1^), graphene has been extensively explored for a wide range of applications (Fig. 2 and 3).^4–7^ In particular, its combination of high surface accessibility and efficient charge transport makes it highly attractive for electrochemical energy storage systems.

**Fig. 2:**
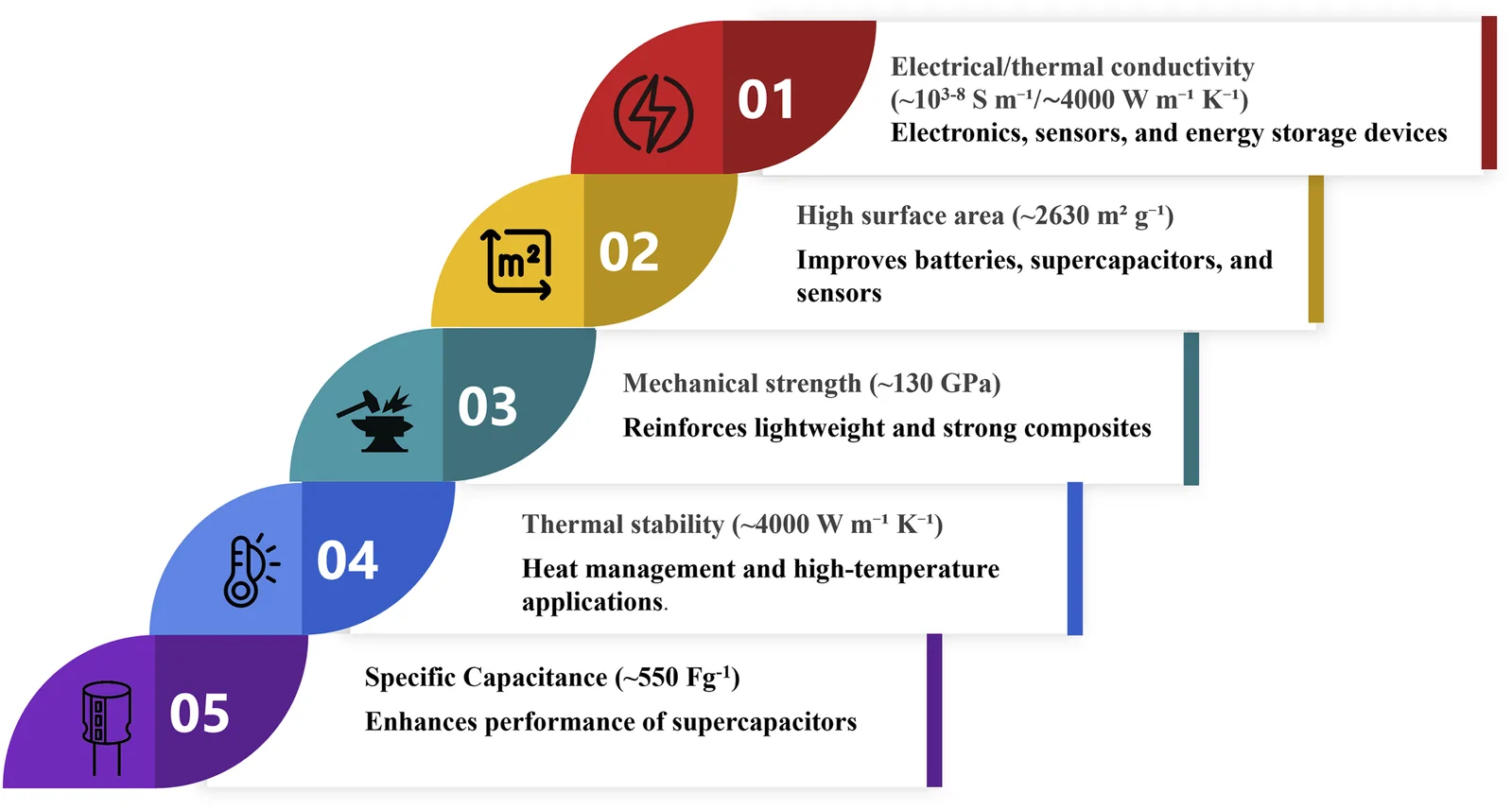
Properties of graphene.

**Fig. 3:**
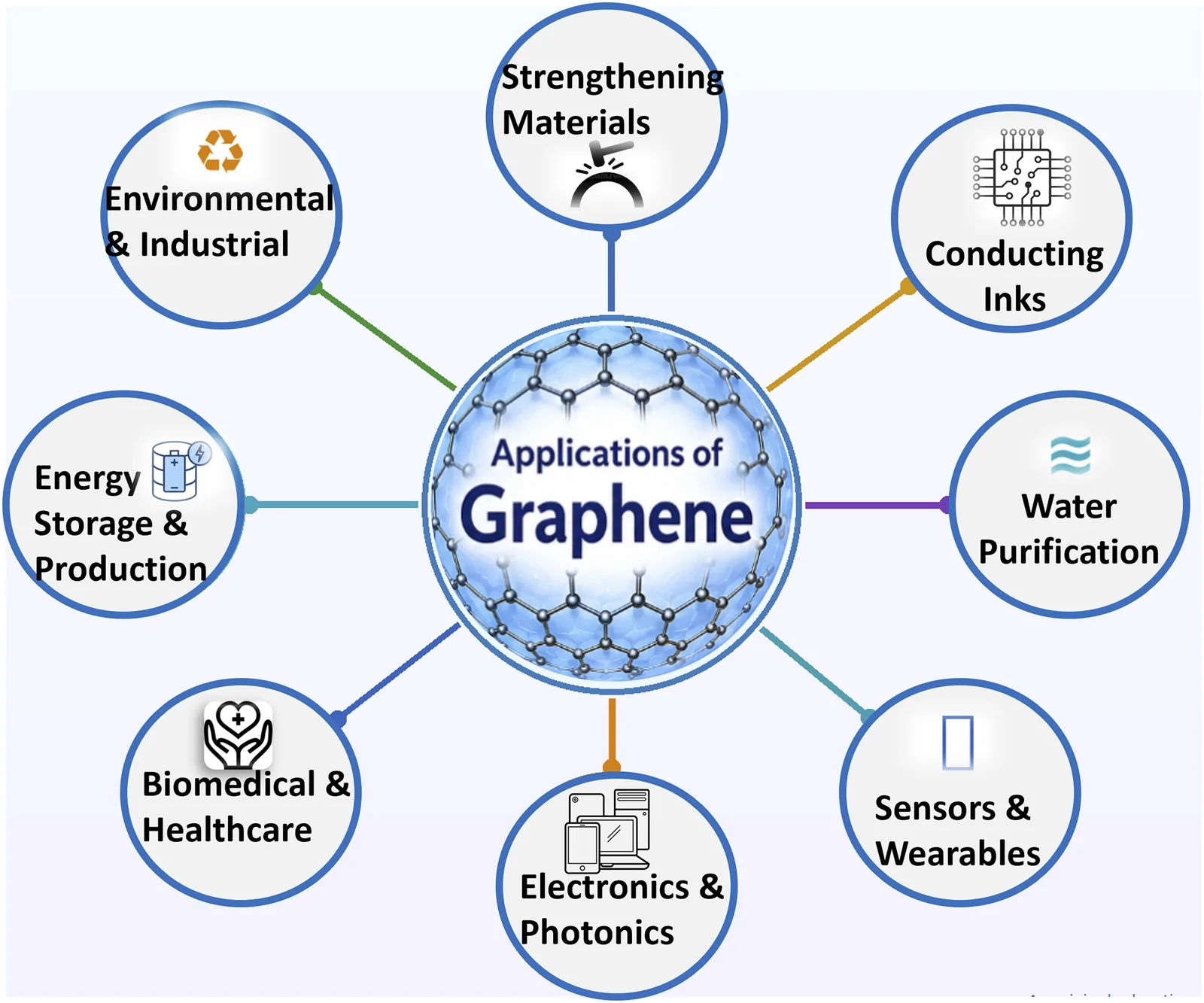
Prominent applications of graphene (figure refined using Perplexity AI for illustrative purposes).

Despite these advantages, the transition of graphene from laboratory-scale research to industrial deployment remains a persistent challenge. A central issue lies in achieving cost-effective, scalable production while maintaining structural integrity, particularly the preservation of the extended sp^2^ carbon bonded π-conjugative network that governs its electronic properties. Conventional synthesis strategies often involve a trade-off between production efficiency and structural quality, with defect generation, especially within the basal plane, emerging as a critical limitation.

Top-down methods involve the progressive exfoliation of bulk carbon materials (especially graphite), into thinner sheets, including mechanical and liquid-phase exfoliation, electrochemical techniques, intercalation-assisted separation, chemical reduction of graphene oxide (GO), and longitudinal unzipping of carbon nanotubes (CNTs).^8,9^ While most of these approaches are generally scalable, they frequently introduce structural defects or require harsh chemical treatments, which can disrupt the conjugated carbon framework.

Bottom-up approaches, in contrast, construct graphene from atomic or molecular carbon precursors.^10^ Representative methods include chemical vapour deposition (CVD), arc discharge synthesis, thermal decomposition of silicon carbide (SiC), and high-temperature conversion of solid carbon sources (Fig. 4). These techniques offer superior control over crystallinity and thickness but typically demand high energy input, specialized infrastructure, and stringent processing conditions.

**Fig. 4:**
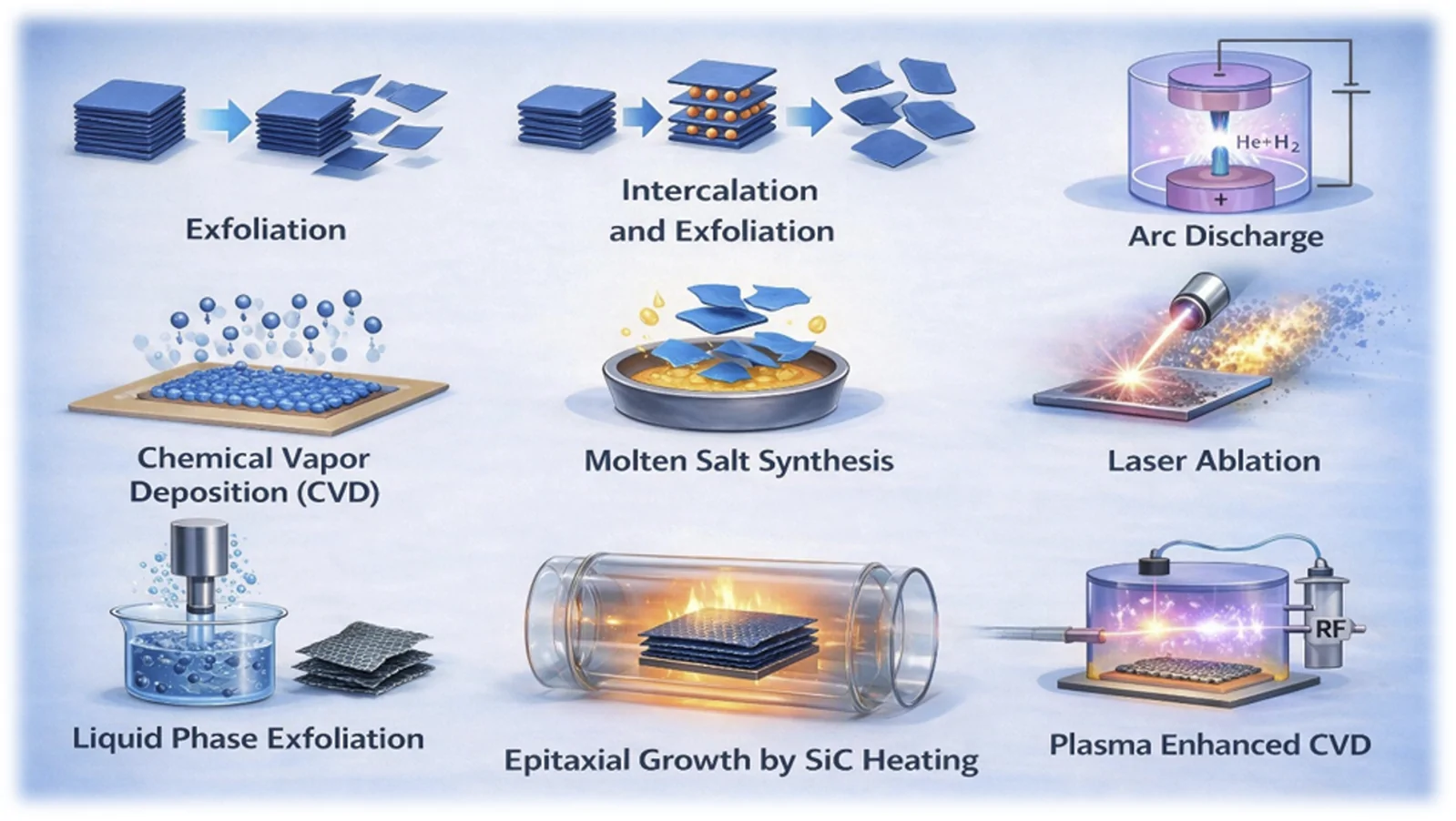
Different synthesis routes for preparing graphene (figure refined using Perplexity AI for illustrative purposes).

The selection of an appropriate synthesis route is therefore governed by a balance between scalability, cost, environmental impact, and the preservation of intrinsic material properties. For instance, mechanical exfoliation of graphite yields high-quality graphene with minimal defects but is inherently limited in throughput.^11^

Liquid-phase exfoliation improves scalability but often relies on energy-intensive processes and solvent handling.^12^ Intercalation-based methods enhance layer separation efficiency but introduce chemical complexity and waste management challenges.^13^ Similarly, the widely adopted GO-to-reduced graphene oxide (rGO) route enables bulk production (Fig. 5, graphite is illustrated as a single layer in the schematic for structural clarity and ease of visualization), yet the oxidative treatments involved significantly disrupt the basal-plane π-conjugated network, even when partial restoration is attempted during reduction.^14^ The unzipping of carbon nanotubes (CNTs) yields structurally controlled graphene nanoribbons, though the high cost of CNT precursors and the oxidative or plasma-based treatments involved limit its commercial scalability.^9^

**Fig. 5:**
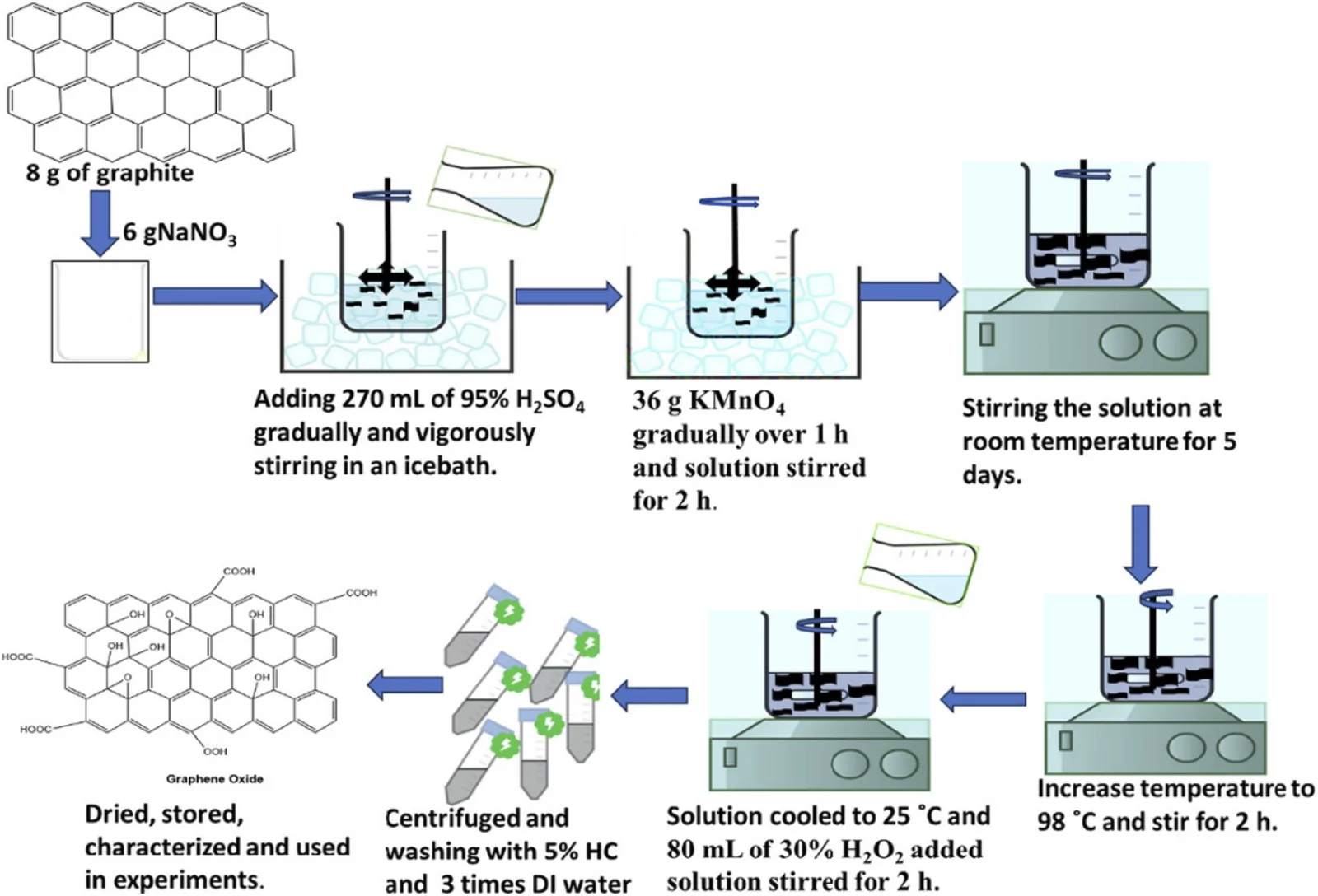
Graphene oxide synthesis route, reproduced from ref. 15 with permission from American Chemical Society,^15^ Copyright 2024.

Bottom-up strategies present a different set of trade-offs. CVD (Fig. 6(a–c)) enables wafer-scale graphene growth with high structural precision, making it suitable for electronic applications; however, it involves high-purity precursors, controlled atmospheres, and substantial energy consumption.^16^ Arc discharge and related methods can produce highly crystalline materials but are energy-intensive and difficult to scale uniformly.^17^ Thermal decomposition of SiC yields device-grade graphene but is restricted by the high cost of substrates and extreme processing conditions.^18^ Other high-temperature carbon conversion routes, while comparatively simpler, still face challenges related to energy demand, emission control, and reproducibility.

**Fig. 6:**
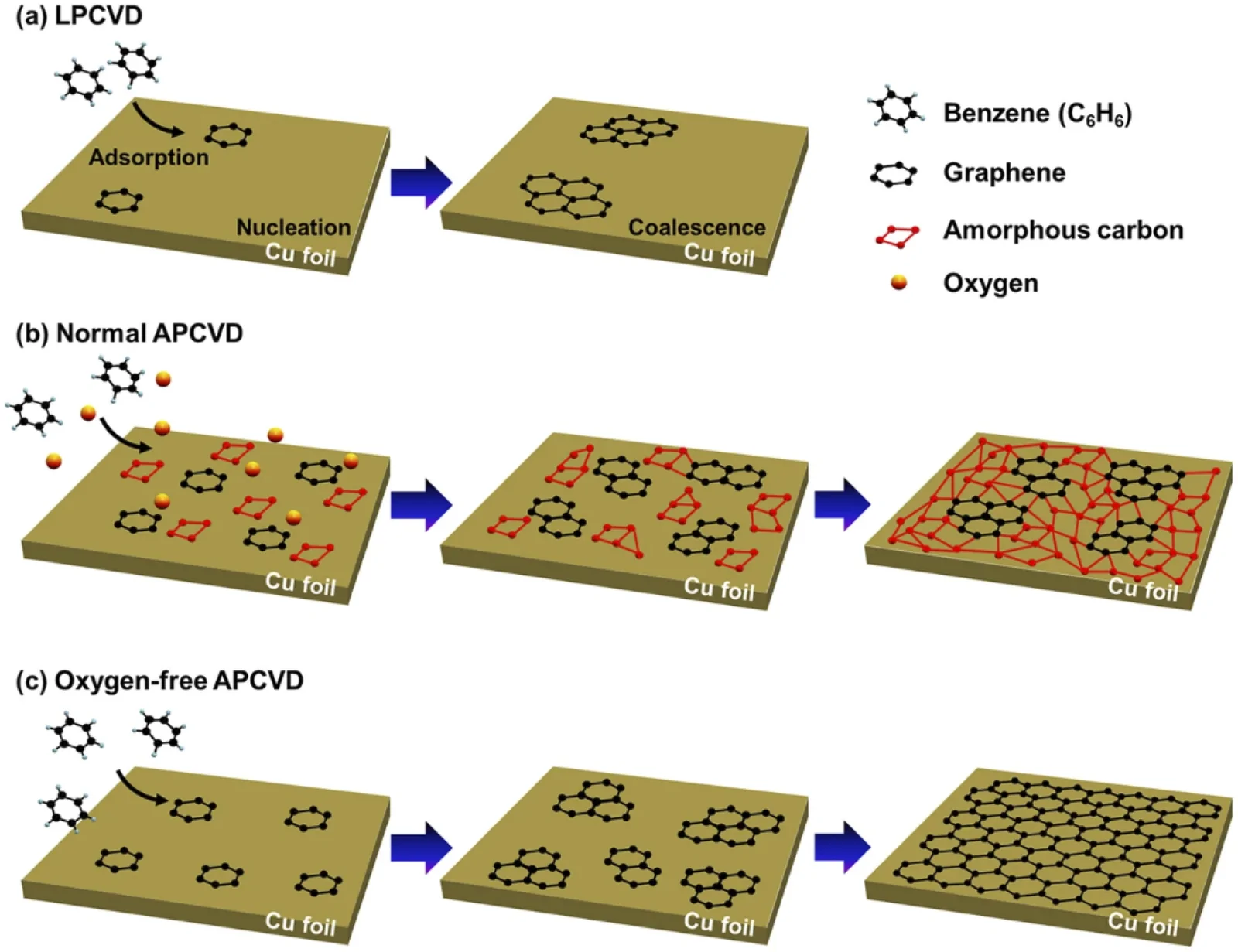
(a–c) Synthetic route of CVD graphene in different conditions, reproduced from ref. 19 with permission from Macmillan Publishers Limited,^19^ Copyright 2015.

Overall, the commonly used synthesis pathways (Table 1) highlight a fundamental gap: the lack of a scalable approach that simultaneously ensures structural integrity, controlled functionalization, cost-effectiveness, and environmental sustainability. Addressing this gap requires strategies that minimize basal-plane disruption while enabling targeted chemical modification. In this context, mechanochemical approaches, particularly shear-assisted ball milling, offer a promising alternative by enabling controlled exfoliation with preferential edge activation, thereby preserving the electronic framework of graphene while introducing functional versatility. Fig. 7 illustrates different graphene systems, including edge carboxylic acid-functionalized graphene.

**Fig. 7:**
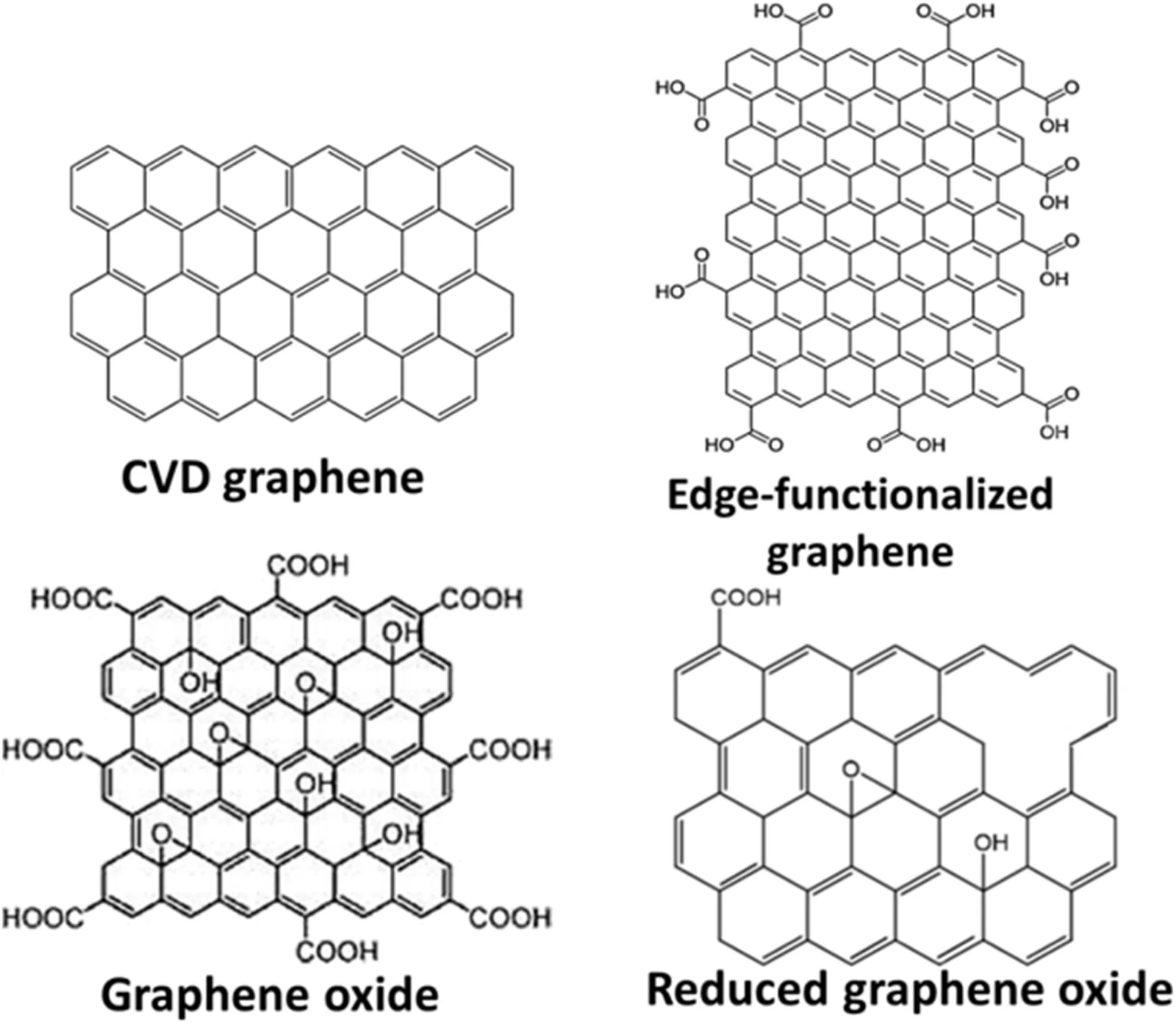
Structure of CVD graphene, edge-functionalized graphene, GO, and rGO, adapted from ref. 20 with permission from Elsevier B.V.,^20^ Copyright 2024.

**Table 1:** Summary of graphene production methods highlighting the nature of graphene produced, its functionalities and defect density

Sl no.	Preparation method	Nature of graphene	Functional groups	Defect density (*I*_D_/*I*_G_ value)	Ref.
1	Electrochemical exfoliation	Multi-layer graphene	C–OH, C–O–C, and O–C=O	0.19	21
2	Plasma-enhanced chemical vapor deposition method	Multi-layer graphene	—	0.96	22
3	Ball milling using sodium sulphate	Few-layer graphene	C–OH, C=O, and C–O	1.00	23
4	Chemical oxidation followed by microwave-assisted reduction	Few-layer graphene	C–OH, C=O, and C–O	2.51	24
5	Coke pyrolysis	Few-layer graphene	C–OH, C=O, C–O, and C–O=C	0.67	25
6	Liquid phase exfoliation	Graphene nanoplatelets	Nonpolar functional groups	0.27	26
7	Chemical oxidation reduction	Multi-layered	C–OH, C=O, C–O, and C–O=C	1.78	27
8	Ball milling using recycled graphite	Graphene nanoplates	Oxygen-containing functional groups	0.12	28
9	Pyrolysis	Few-layer graphene	Phenol and aromatic functional groups	0.6	29
10	Suspension electrolysis	Few-layer graphene	C–O–C, C–O, C=O, and C–C	0.04	30

While graphene synthesis, mechanochemical processing, defect engineering, and graphene-based energy storage have each been the subject of numerous reviews, the specific role of shear-driven ball milling in producing edge-functionalized graphene (EFG) has received comparatively limited attention. This review brings together current advances in shear-assisted exfoliation, edge-selective functionalization, and the influence of milling agents on graphene chemistry. Particular emphasis is placed on the characterization approaches used to differentiate edge and basal-plane modifications and on understanding how these structural features govern electrochemical performance. The review further examines the relationships between processing conditions, defect characteristics, surface functionalities, and energy-storage behaviour, while also considering practical aspects such as scalability, sustainability, economic feasibility, and industrial implementation. By presenting these topics within a single framework, this article aims to provide a comprehensive understanding of shear-driven ball milling as a versatile route for the development of advanced graphene materials specifically targeting energy-storage applications.

## 2. Mechanical exfoliation *via* shear-driven ball milling of graphite

Shear-driven ball milling is a solid-state mechanochemical process in which mechanical energy is transferred to graphite through repeated ball–powder–wall and ball–powder–ball interactions.^31^ Unlike impact-dominated milling, shear-governed regimes promote interlayer sliding, delamination, and controlled structural reorganization of graphitic stacks.^32^

The efficiency of exfoliation is dictated by stress distribution, collision dynamics, rotational kinematics, and the associated non-equilibrium pathways governing bond rupture and structural stabilization. Importantly, when operated under shear-prevalent conditions, this approach enables layer separation with a limited disruption to the extended sp^2^-bonded framework, forming the basis for producing structurally preserved graphene.

### 2.1. Shear *vs.* impact forces

Two principal stress modes operate during ball milling, the normal impact stress and tangential shear stress.^32^ Impact-dominated regimes typically arise at higher rotational speeds and lower ball filling ratios, where balls undergo cataracting motion and collide with high kinetic energy. These collisions generate localized compressive stresses that promote particle fracture, lattice distortion, and eventual amorphization rather than controlled exfoliation.^33^

In contrast, shear-dominated regimes occur under cascading motion, where balls slide relative to one another and along the jar wall. This generates tangential forces that induce interlayer slippage (Fig. 8) across the weak van der Waals gaps of graphite (∼0.34 nm spacing).^34^ Given the strong in-plane sp^2^ bonding and comparatively weak interlayer interactions in graphite, shear stress is inherently more effective for delamination into few-layer graphene. Crucially, this mode of stress application confines structural modification primarily to layer separation and edge formation, thereby minimizing disruption to the basal-plane π-conjugated carbon network.

**Fig. 8:**
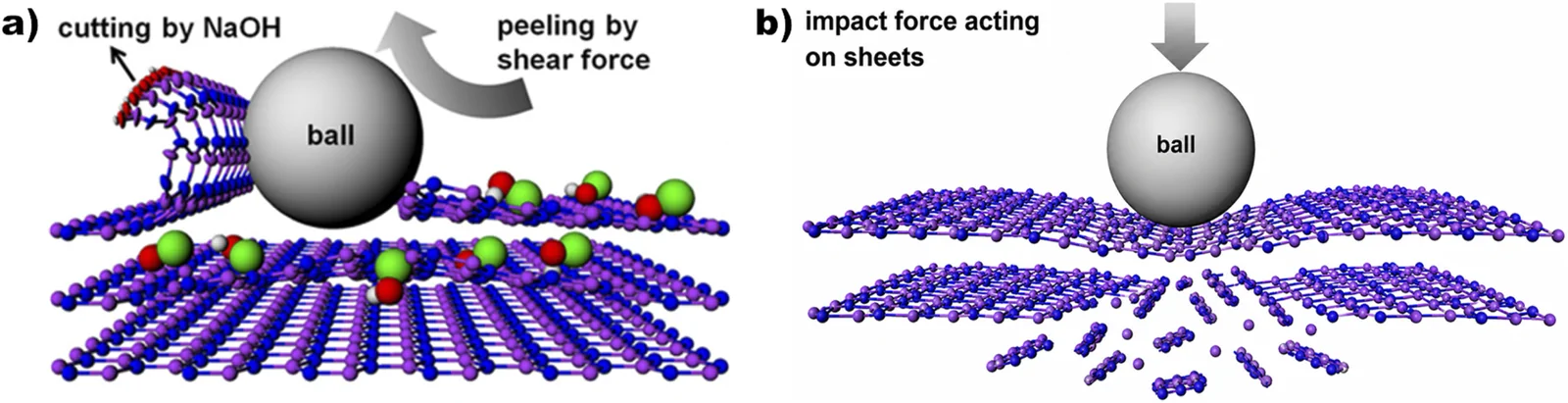
(a) Representation of graphite layer exfoliation by shear forces during ball milling, reproduced from ref. 35 with permission from American Chemical Society,^35^ Copyright 2015. (b) Representation of impact forces in ball milling.

### 2.2. Energy transfer mechanisms

Mechanical energy is introduced through the rotational motion of the milling system and is transferred *via* ball–ball collisions, ball–powder interactions, and ball–wall frictional contact. The kinetic energy of the milling media depends on the rotational speed, jar dimensions, and mass distribution.^36^ Upon interaction, this energy is dissipated through a combination of plastic deformation, interlayer cleavage, localized transient heating, and lattice strain generation.^37^ In shear-governed regimes, a larger fraction of the input energy is converted into frictional work rather than impulsive impact. This promotes progressive interlayer sliding and controlled delamination instead of catastrophic lattice breakdown. Consequently, energy dissipation pathways favor structural rearrangement and limited bond perturbation, rather than extensive bond rupture or rehybridization.^38^ This distinction is critical in maintaining the electronic continuity of graphitic domains during exfoliation.

### 2.3. Role of rotational speed and milling time

Rotational speed governs the transition between cascading, cataracting, and centrifuging regimes. At low speeds, insufficient energy transfer limits effective exfoliation.^39^ At intermediate speeds, shear forces dominate, enabling efficient layer separation with controlled structural modification. At excessively high speeds, impact contributions increase, leading to elevated defect densities and potential amorphization. The milling time determines the cumulative energy input. Short durations result in partial exfoliation, while moderate durations promote progressive thinning and edge development. Prolonged milling, however, can induce excessive fragmentation, structural disorder, and environmental degradation effects (*e.g.*, oxidation under ambient conditions).^40^ Therefore, optimization of speed-time combinations is essential to maximize shear-induced exfoliation while suppressing unnecessary lattice damage.

### 2.4. Defect generation mechanisms

Mechanical activation inevitably introduces structural defects, including edge formation, vacancy defects, Stone–Wales transformations, and limited sp^2^-to-sp^3^ rehybridization under high stress conditions.^41,42^ However, the nature and distribution of these defects are strongly dependent on the dominant stress mode. Shear-prevalent conditions favor the generation of edge-localized defects associated with layer separation, whereas impact-dominated conditions increase the bulk defect density and amorphous carbon formation. This distinction is central to defect engineering, whereas under controlled shear, defect introduction is spatially confined and structurally selective, enabling the retention of extended graphitic domains. Such controlled defect topology forms the basis for subsequent chemical modification without compromising the intrinsic electronic framework.

### 2.5. Thermodynamic considerations

Graphite exfoliation requires overcoming interlayer van der Waals interactions (∼2–2.5 kJ mol^−1^ per carbon atom pair).^43^ Mechanical activation provides the necessary energy to overcome these interactions, driving the system into a non-equilibrium state. Key thermodynamic features include strain-induced increases in Gibbs free energy, entropy gain upon layer separation, and localized transient temperature rises that facilitate bond rearrangement and defect stabilization.^44,45^ Although graphene is thermodynamically less stable than bulk graphite under equilibrium conditions, shear-driven mechanical activation enables access to metastable few-layer structures by continuously perturbing the system away from equilibrium.^46^ Rapid dissipation of energy prevents complete structural relaxation, thereby stabilizing exfoliated configurations.

Overall, shear-driven ball milling represents a non-equilibrium, moderate-energy strategy for graphite exfoliation that emphasizes controlled stress application and energy dissipation. By selectively promoting interlayer sliding over lattice fracture, this approach enables the production of structurally preserved, low-defect graphene with preferential edge activation. Such control over defect topology and structural integrity provides a mechanistic foundation for subsequent chemical functionalization and application-specific material design.^39^

## 3. Control strategies for regulating exfoliation and surface chemistry

Precise regulation of graphite exfoliation in shear-assisted ball milling requires coordinated control over milling agents, process parameters, and the chemical environment.^47^ Building on the mechanistic framework established in Section 2, this section focuses on how these variables govern structural evolution and surface chemistry, with particular emphasis on achieving edge-selective modification while retaining the integrity of graphitic domains.^48,49^

### 3.1. Milling agents of graphite

Milling agents play a dual role by influencing both stress transfer and chemical interactions during exfoliation.^50^ They regulate interlayer separation, sheet size, and the nature of surface functionalities introduced during processing.^51^ Based on their chemical characteristics, they can be broadly categorized as discussed in the following sections.

#### 3.1.1 Inert solid milling agents

Inert agents such as alkali halide salts, carbonate salts, and inorganic solid spacers primarily act as physical separators that suppress restacking and enhance shear transmission (Fig. 9).^52^ These systems are particularly effective when the objective is to obtain few-layer graphene with minimal basal-plane perturbation. The absence of strong chemical reactivity allows exfoliation to proceed without significant alteration of the graphene's in-plane π-conjugative network.

**Fig. 9:**
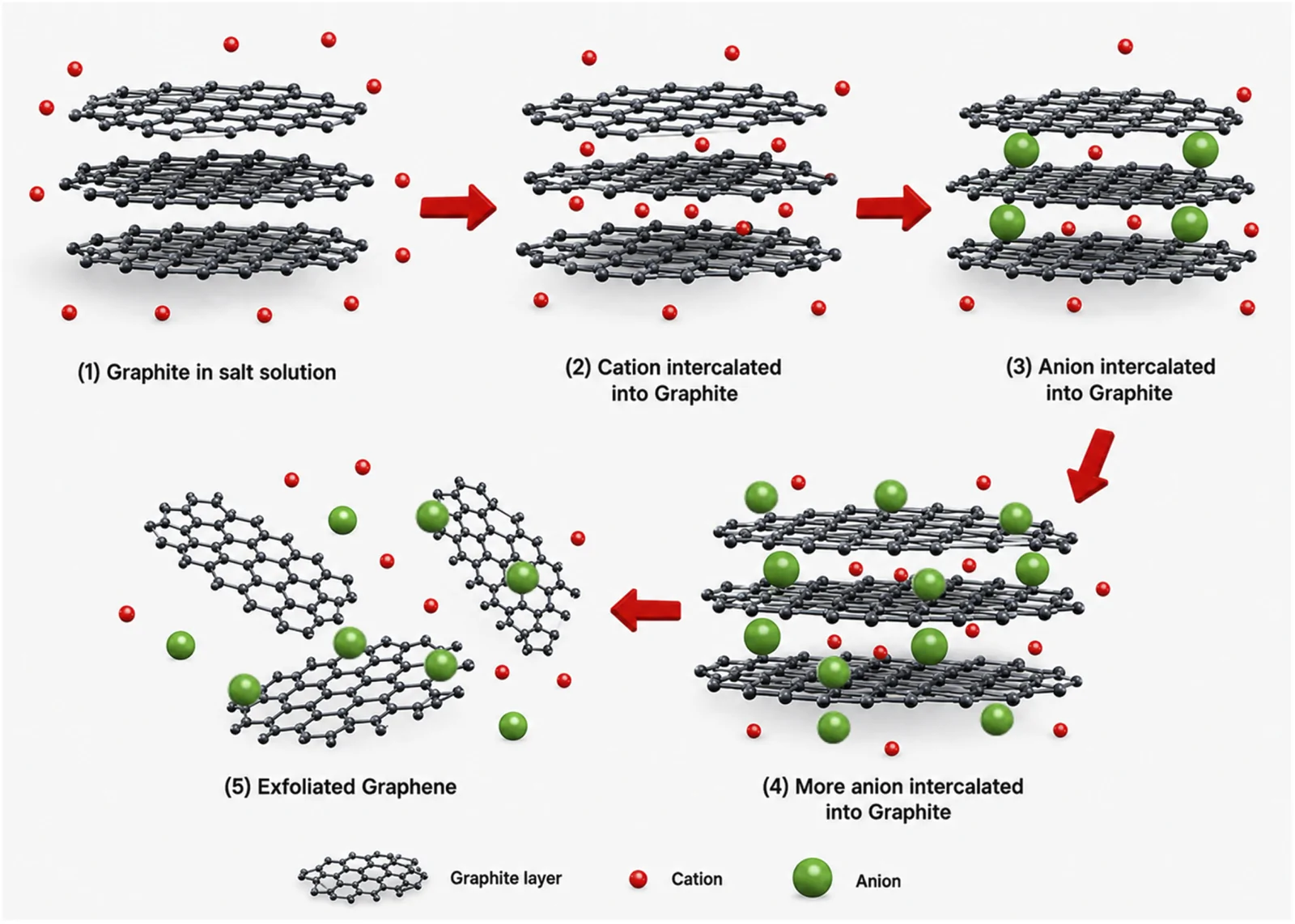
Inorganic salt assisted ball milling, adapted from ref. 57 with permission from The Royal Society of Chemistry,^57^ copyright 2017.

For instance, Arao *et al.* employed K_2_CO_3_-assisted milling followed by sonication to obtain edge-activated graphite, exhibiting improved dispersibility in polar solvents.^53^ Similarly, NaCl-assisted graphite exfoliation has been shown to yield well-dispersed graphene with sharp edges and controlled structural features.^54^ Variations in salt type influence exfoliation efficiency and layer ordering, highlighting the role of ionic size and interaction strength in modulating interlayer separation.^55^ The water solubility of these agents further enables straightforward post-processing with minimal contamination. Alam and co-workers investigated alkali halide salt-assisted graphite exfoliation using NaCl, KCl, and LiI.^56^ After salt removal, predominantly defect-free single-layer graphene was obtained. In comparison, samples synthesized with different salts displayed variations in exfoliation and structural ordering, which highlights the influence of the milling agents.

#### 3.1.2 Reactive solid milling agents

Reactive agents such as urea, melamine, and thiourea enable simultaneous exfoliation and heteroatom incorporation through mechanochemically induced reactions. Under milling conditions, these compounds generate reactive intermediates that preferentially interact with defect and edge sites, enabling localized chemical modification. Urea-assisted milling, for example, facilitates nitrogen doping while maintaining structural continuity, yielding graphene with enhanced electrochemical performance.^58^ Here, urea plays a dual role as a solid doping agent and an exfoliating aid. Similarly, combined systems such as NaCl-melamine provide a balance between physical separation and chemical doping, resulting in controlled nitrogen incorporation (∼5 wt%) without extensive structural degradation and delivered a specific capacity of 582 mAh g^−1^ as a battery electrode material.^59^

Dual-doping strategies using sulfur- and nitrogen-containing precursors further illustrate the tunability of this approach. Lu and his team synthesized dual-atom-doped carbon materials using corn starch and thiourea (CH_4_N_2_S) as the precursors and were able to get a specific capacity of 400 mAh g^−1^ for sodium ion batteries.^60^ The heteroatom doping enhances electronic conductivity by providing additional charge carriers, improve surface wettability with electrolytes, and offers more active sites for energy storage.^61^ While increased reactivity enhances the functionalization efficiency, excessive chemical interaction can introduce additional disorder. Therefore, controlled selection and proportioning of reactive agents are essential to maintain the balance between chemical modification and structural preservation.

#### 3.1.3 Carbohydrate-based edge-centered milling agents

Carbohydrates and their derivatives (*e.g.*, glucose, sucrose, starch, sorbitol, ethyl cellulose, *etc*.) represent a class of sustainable milling agents that combine mild exfoliation with controlled surface modification.^62–65^ Their hydroxyl-rich structures facilitate interlayer separation through hydrogen bonding while acting as compliant spacers under shear. During milling, partial dehydration or mild carbonization can introduce oxygen-containing functionalities in a controlled manner without inducing aggressive oxidation. In addition, non-conventional hydrogen bonding interactions between C–H/O–H groups of carbohydrates and the aromatic π-system of graphite contribute to effective exfoliation and stabilization of separated layers (Fig. 10).^66^ Compared to strongly oxidative systems, carbohydrate-assisted approaches typically yield graphene with moderate defect densities and improved structural retention.

**Fig. 10:**
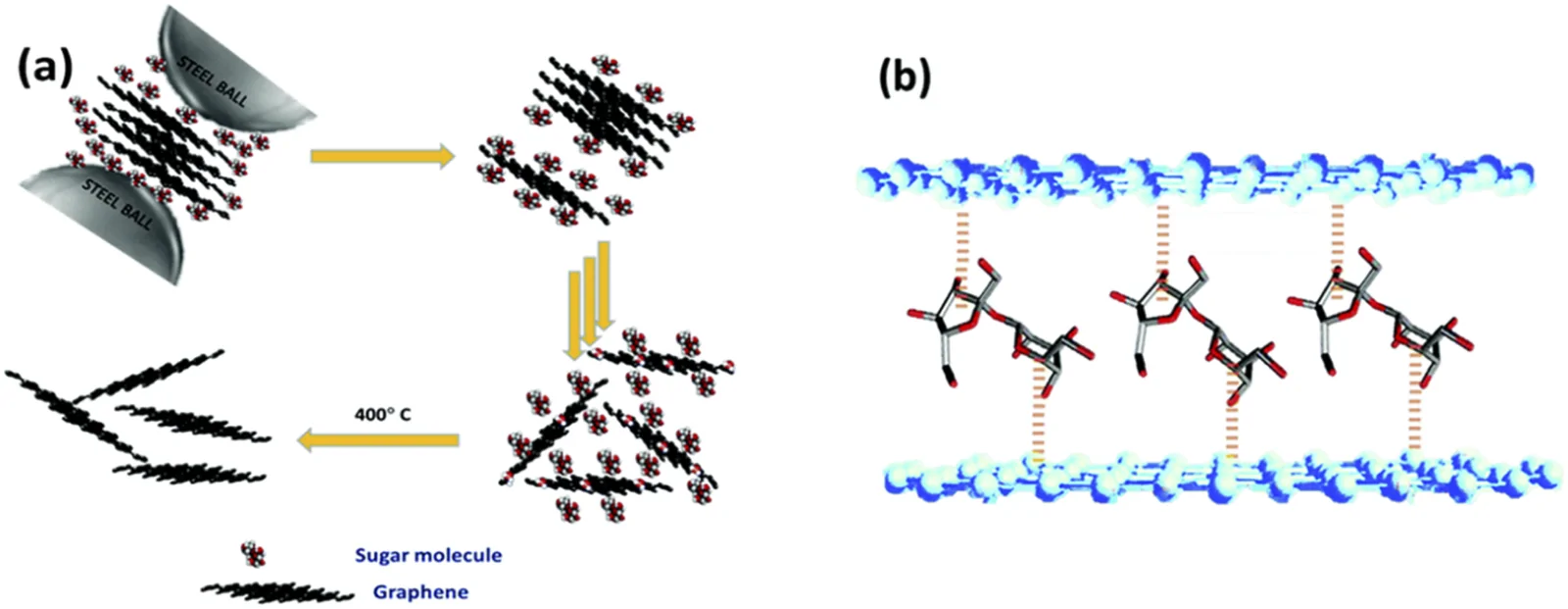
(a) Carbohydrate assisted ball milling and (b) mechanism of exfoliation, reproduced from ref. 56 with permission from The Royal Society of Chemistry,^66^ copyright 2017.

#### 3.1.4 Liquid-assisted exfoliating agents

Liquid-assisted systems utilize solvents to modulate interlayer interactions and facilitate shear-driven exfoliation.^12^ Solvent properties, including surface tension, polarity, and intermolecular interaction parameters, govern exfoliation efficiency and dispersion stability (Fig. 11).^67^ Wet ball milling extends this concept by enabling exfoliation directly within a liquid medium. For example, exfoliation in *N*,*N*-dimethylformamide (DMF) yields few-layer graphene with preserved electrical conductivity.^68^ The resulting supernatant contained irregular single- and few-layer graphene (≤3 layers, ∼0.8–1.8 nm thick) with an electrical conductivity of ∼1.2 × 10^3^ S m^−1^ at room temperature. Variations in solvent chemistry influence the layer thickness, crystallite size, and defect levels, as observed in systems employing kerosene or 2-ethylhexanol.^69^ Graphene exfoliated in 2-ethylhexanol exhibits more layers, larger crystal size, and fewer defects, whereas exfoliation in kerosene produces fewer layers, smaller crystal size, and higher defect density. Additionally, reactive solvent systems such as maleic anhydride in NMP enable concurrent exfoliation and functionalization, improving dispersibility without extensive lattice disruption.^70^

**Fig. 11:**
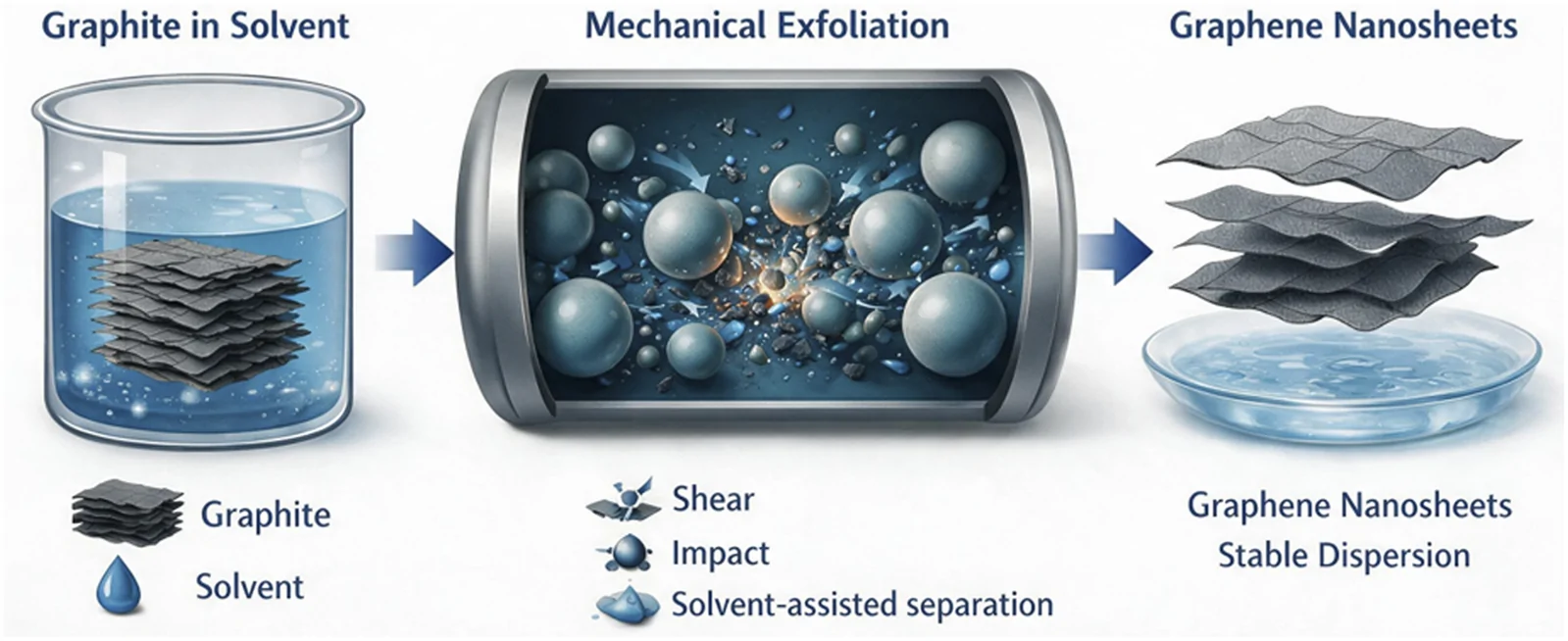
Liquid-assisted ball mill exfoliation of graphite (Figure generated using Perplexity AI for illustrative purposes).

However, prolonged milling in liquid environments may lead to solvent decomposition or unintended functionalization, necessitating careful optimization of processing conditions.

### 3.2. Atmosphere control

The milling atmosphere serves as a critical parameter in regulating surface chemistry. Inert environments (*e.g.*, Ar, N_2_, *etc*.) suppress oxidative reactions and restrict chemical modification primarily to mechanically generated sites.^71^ This is particularly important for preserving electronic transport characteristics. In contrast, controlled exposure to oxygen or moisture can introduce specific functional groups in a regulated manner without the need for harsh chemical treatments.^72^ However, uncontrolled atmospheric conditions accelerate defect accumulation and structural disorder. Thus, atmosphere control provides a complementary pathway for tuning surface chemistry alongside milling agent selection.

### 3.3. Milling duration

Exfoliation proceeds through a time-dependent structural evolution governed by the cumulative energy input and chemical environment (Fig. 12). Initial milling stages involve particle refinement and onset of interlayer sliding with minimal structural disruption. At intermediate durations, shear-driven delamination reaches an optimum, yielding few-layer graphene with balanced edge activation and structural integrity.^73^ Prolonged milling leads to increased defect accumulation, fragmentation, and potential environmental degradation effects.^74^ In reactive systems, extended durations also enhance functional group incorporation, which may exceed desirable levels. The optimal milling duration therefore depends strongly on the nature of the milling agent: inert systems tolerate longer processing, whereas reactive systems require tighter temporal control.

**Fig. 12:**
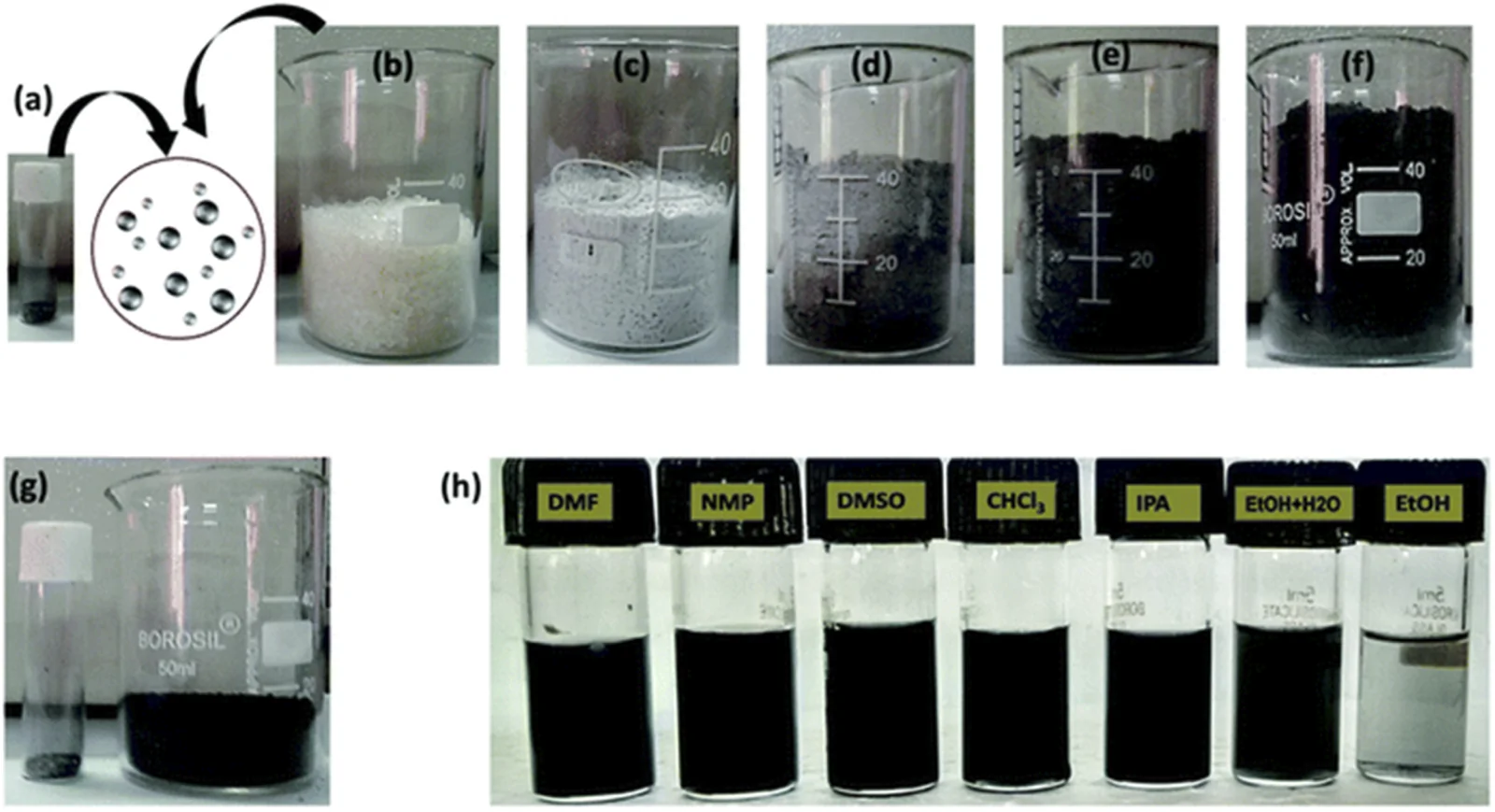
(a) Graphite, (b) sucrose, (c–f) ball-milled mixture after 2, 10, 20, and 30 h, (g) 0.2 g of graphite (left) along with 0.2 g of 30 h ball-milled mixture (right), and (h) its dispersibility in various solvents, reproduced from ref. 56 with permission from The Royal Society of Chemistry,^66^ copyright 2017.

### 3.4. Ball-to-powder ratio

The ball-to-powder ratio (BPR) governs the collision frequency and stress intensity.^75^ Higher BPR values not only increase the frequency of shear events, improving the exfoliation efficiency, but may also introduce unwanted impact contributions if excessively high. Conversely, lower BPR values reduce structural damage but may limit the exfoliation yield. An optimized BPR ensures effective stress transfer while maintaining shear-dominant conditions. The reported values typically range from 100 : 1 to 1 : 1, depending on system configuration and desired outcomes.^75–77^

### 3.5. Post-treatment strategies

Post-processing plays a crucial role in refining the structural and chemical characteristics of the exfoliated material. Thermal annealing can remove weakly bound species, reduce residual oxygen content under inert conditions, and partially restore graphitic ordering.^78^ Chemical washing eliminates residual milling agents, including salts and organic additives. Mild reduction or thermal treatments can further tune surface chemistry by selectively removing excessive functional groups while retaining beneficial edge functionalities.^79,80^ Such treatments enhance the electrical conductivity and structural stability without negating the advantages of controlled functionalization.

Effective regulation of graphite exfoliation requires a synergistic combination of milling agent selection, process parameter optimization, and chemical environment control. Across diverse systems, maximum exfoliation efficiency is consistently achieved under shear-dominant, chemically moderate conditions, where structural modification remains spatially controlled. This enables the production of graphene with tailored defect distribution and surface chemistry while preserving the continuity of graphitic domains, providing a foundation for subsequent functional and application-specific optimization.

## 4. Structure evolution during ball milling of graphite: mechanisms, metrics, and characterization strategies

Understanding the structural evolution of graphite during ball milling is essential for correlating processing conditions with exfoliation efficiency and material quality. As outlined in earlier sections, mechanochemical treatment induces progressive transformations including layer delamination, lateral size reduction, defect generation, and surface modification. These changes directly influence the layer thickness, flake dimensions, crystallinity, and porosity of the resulting graphene materials.^81,82^ A systematic evaluation of these structural parameters is therefore necessary to establish clear process–structure relationships.

### 4.1. Milling agent aided exfoliation mechanism

Structural evolution during ball milling reflects the gradual transformation of bulk graphite into few-layer graphene through shear-governed delamination. Progressive milling leads to the thinning of graphite platelets, accompanied by the formation of new edge sites and partial reduction in stacking order. The presence of milling agents plays a stabilizing role by interacting with these newly generated surfaces, thereby limiting restacking and preserving exfoliated configurations. This stabilization is particularly important in maintaining separated layers during both milling and subsequent handling. Milling agent-aided ball mill exfoliation is already discussed in section 3.1 and described in Fig. 10.

Experimental observations consistently indicate that, under controlled conditions, exfoliation proceeds without extensive disruption of the basal graphitic framework. Electron microscopy reveals increasingly transparent and thinner sheets relative to bulk graphite, while diffraction features show gradual broadening of the X-ray diffraction peak that originated from the (002) plane, indicating reduced stacking coherence rather than complete structural collapse.^83^ These signatures collectively point toward controlled delamination with retention of extended graphitic domains.

### 4.2. Layer number control

Controlling the number of graphene layers is a key indicator of exfoliation efficiency. Thickness reduction occurs progressively with milling, transitioning from multilayer graphite to few-layer graphene under optimized conditions.^39,74^ Direct measurement techniques such as atomic force microscopy enable statistical determination of layer thickness through height profiling, while high-resolution electron microscopy provides localized confirmation *via* edge fringe analysis.^82^ The ability to achieve few-layer structures without excessive fragmentation reflects a balance between energy input and structural preservation. Spectroscopic signatures (Section 5.2) further support layer number estimation.^84^

### 4.3. Lateral size reduction

Mechanical processing inherently leads to a reduction in lateral flake dimensions due to progressive fragmentation of graphite platelets.^85^ Electron microscopy is commonly employed to assess the changes in morphology and size distribution before and after milling,^82^ while bulk particle analysis techniques provide complementary statistical insights.^86^ With increasing milling duration, the system evolves toward a broader distribution of flake sizes, comprising both partially exfoliated larger sheets and smaller fragments.^74^ Maintaining an optimal lateral dimension is critical, as excessively small flakes can compromise structural continuity, whereas larger sheets are less favorable for efficient electrolyte diffusion. Controlled processing therefore aims to balance delamination and fragmentation.

### 4.4. Structural disorder and amorphisation risk

While exfoliation requires mechanical activation, excessive energy input can lead to progressive structural disorder. Prolonged milling may introduce lattice distortions and reduce crystallinity, particularly under conditions where impact contributions become significant.^33^ Diffraction-based measurements typically reveal this transition through peak shifting and reduction in crystallite dimensions,^83^ while spectroscopic analysis (Section 5.2) provides further insight into defect evolution. Importantly, under shear-controlled conditions, structural modification remains largely confined to edge regions, allowing substantial portions of the graphitic framework to remain intact. This highlights the importance of maintaining processing parameters within an optimal window to avoid unnecessary degradation.

### 4.5. Pore formation

Mechanochemical processing can also induce the formation of micro- and mesoporous features within graphene structures.^87^ Pore generation arises from a combination of edge formation, fragmentation of graphitic domains, and removal of milling agents during post-treatment.^79^ Surface area analysis using N_2_ adsorption techniques often shows increased pore volume and surface accessibility following milling.^88^ In systems involving organic additives, thermal removal can generate additional porosity through gas evolution, leading to perforated sheet structures.^78^ While moderate pore formation enhances accessibility, excessive porosity may indicate structural overprocessing and loss of conductive pathways.

It is noteworthy that conventional BET analysis may underestimate the accessible surface area of graphene due to limited adsorption on open two-dimensional surfaces and agglomeration effects.^65,89^ Alternative approaches, such as methylene blue dye adsorption methods, can provide complementary insights into effective surface accessibility.^90^

### 4.6. Kinetic perspective of graphite exfoliation

The structural transformation of graphite during ball milling can be described using kinetic frameworks that relate exfoliation progress to mechanical processing conditions.^91,92^ In this context, each collision event contributes incrementally to structural evolution, and exfoliation proceeds as a cumulative process. The extent of transformation is often treated as a function of milling time, with measurable parameters such as layer thickness, surface area, or crystallinity serving as indicators of progression.^74,93^ However, because collision frequency and stress intensity vary across systems, expressing exfoliation in terms of cumulative mechanical energy input provides a more generalized description.^94,95^ Such kinetic approaches enable the identification of processing regimes, where exfoliation efficiency is maximized, while structural degradation is minimized. This provides a quantitative basis for optimizing milling parameters and improving reproducibility in graphene production.

## 5. Functionalization/structure-morphology investigation and edge *vs.* basal plane discrimination

In mechanochemical graphene synthesis, functionalization is intrinsically coupled with exfoliation, particularly in the presence of reactive milling agents. Unlike post-synthetic modification strategies, ball milling enables *in-situ* chemical interactions during layer separation, resulting in simultaneous structural transformation and chemical incorporation.^58,59^ However, accurate identification of functional groups and, critically, their spatial distribution within the graphene framework requires an integrated, multi-technique analytical approach. This section establishes a framework for determining functionalization and distinguishing edge-localized functionalization from basal-plane modification using complementary characterization tools.

### 5.1. Chemical state analysis: XPS and FTIR spectroscopy

X-ray photoelectron spectroscopy (XPS) is the primary technique for determining the elemental composition and chemical bonding environments in functionalized graphene.^96^ Deconvolution of the C 1s spectrum reveals contributions from sp^2^ and sp^3^ carbons, along with functional groups such as C–O, C=O, C–N, C=N, C–S, and O–C=O.^97^ The relative evolution of these components compared to pristine graphite provides direct evidence of functional group incorporation. The nature of different carbon species and their corresponding binding energies obtained from the deconvoluted C 1s spectrum are listed in Table 2, where M indicates any metal.

**Table 2:** Nature of carbon in the deconvoluted C 1s spectrum and the corresponding binding energies

Nature of carbon	Binding energy
M–C	283.5–284.1 eV
sp^2^ C=C	284.4–284.6 eV
sp^3^ C–C	284.8–285.2 eV
C–O/C–N	285.7–286.6 eV
C=O/C=N	285.7–288.0 eV
O–C=O	288.5–289.0 eV

However, while XPS is highly sensitive to chemical states, its spatial resolution is limited. Techniques such as angle-resolved XPS or depth profiling can provide indirect information regarding surface *versus* subsurface functionalization, but they do not unambiguously distinguish edge from basal-plane modification.^98–100^Fig. 13 shows the structure of EFG along with its representative XPS spectrum, providing information on its functionalities.

**Fig. 13:**
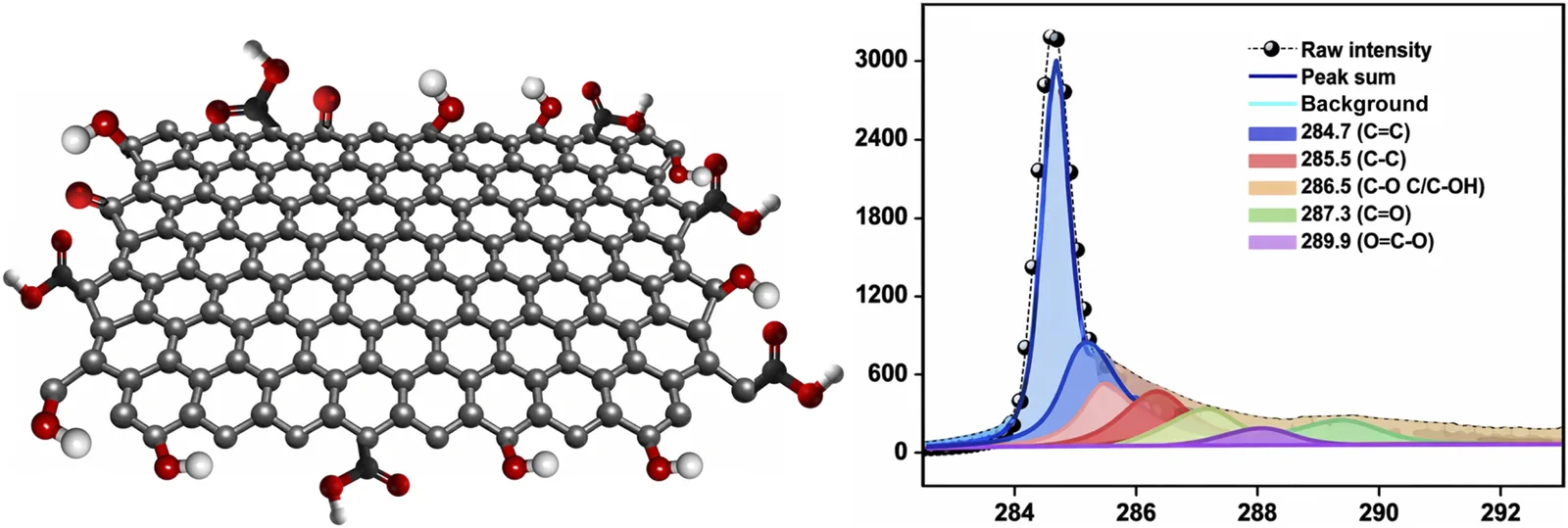
EFG and its representative XPS.

Fourier-transform infrared (FTIR) spectroscopy complements XPS by identifying vibrational signatures of functional groups. Characteristic absorption bands corresponding to O–H (∼3350–3600 cm^−1^), C=O (∼1650–1750 cm^−1^), and C–O (∼1000–1300 cm^−1^) confirm the presence of oxygen-containing species.^101–103^ In mildly functionalized ball-milled systems, these bands can be typically weak yet discernible, indicating limited incorporation of functional groups. Importantly, FTIR does not provide spatial information, but when interpreted alongside XPS, it supports the conclusion of controlled, low-density functionalization rather than extensive oxidation.^65,66^

### 5.2. Defect-sensitive probing: Raman spectroscopy

Raman spectroscopy serves as a critical tool for probing the defect structure and differentiating functionalization pathways.^84^ The key spectral features include the following bands: (a) D band (∼1346 cm^−1^, defect-activated breathing mode), (b) G band (∼1579 cm^−1^, in-plane vibration of sp^2^ carbon), (c) D′ band (∼1620 cm^−1^, intravalley scattering-related feature), and (d) 2D (G′) band (∼2728 cm^−1^, second-order phonon process sensitive to the layer number).^104–106^ The shape and intensity of the 2D band are commonly used to estimate the number of graphene layers, particularly when the sheets are well-exfoliated and not restacked.^107^ The *I*_2D_/*I*_G_, peak intensity ratio serves as a reliable indicator of graphene layer number. It is highest for defect-free monolayer graphene and decreases with increasing number of layers. Typically, bilayer graphene shows values of 1–2, while multilayer graphene exhibits lower ratios (<0.5–1), approaching that of graphite. The *I*_D_/*I*_G_ intensity ratio provides a measure of defect density. In highly oxidized systems such as GO, this ratio approaches ∼1, reflecting extensive lattice disruption.^108,109^ In contrast, ball-milled graphene exhibiting moderate or lower *I*_D_/*I*_G_ values indicates a more controlled defect landscape. More importantly, defect type discrimination can be achieved using the *I*_D_/*I*_D′_ ratio. Lower values (∼3) are associated with edge-dominated defects, whereas higher values (∼3–4) indicate basal-plane defects such as vacancies. Raman mapping further strengthens this distinction by revealing spatial localization of D-band intensity at flake edges, providing direct experimental evidence of edge-selective functionalization. The retention of a relatively well-defined 2D band, albeit broadened, supports the preservation of extended sp^2^ domains, indicating that mechanochemical processing under controlled conditions modifies graphene without complete disruption of the π-conjugated network.^110^

### 5.3. Morphological and local structural analysis

High-resolution imaging techniques provide direct visualization of structural features associated with functionalization (Fig. 14). Electron microscopy enables observation of layer continuity, edge morphology, and defect structures such as holes or edge roughness.^111^ Advanced approaches such as scanning transmission electron microscopy (STEM) combined with electron energy loss spectroscopy (EELS) allow spatially resolved analysis of bonding environments.^112^ Variations in the carbon K-edge can distinguish between sp^2^ and sp^3^ hybridization, offering localized insight into structural modification. These techniques are particularly valuable in confirming that functionalization is predominantly localized at sheet peripheries rather than uniformly distributed across basal planes.

**Fig. 14:**
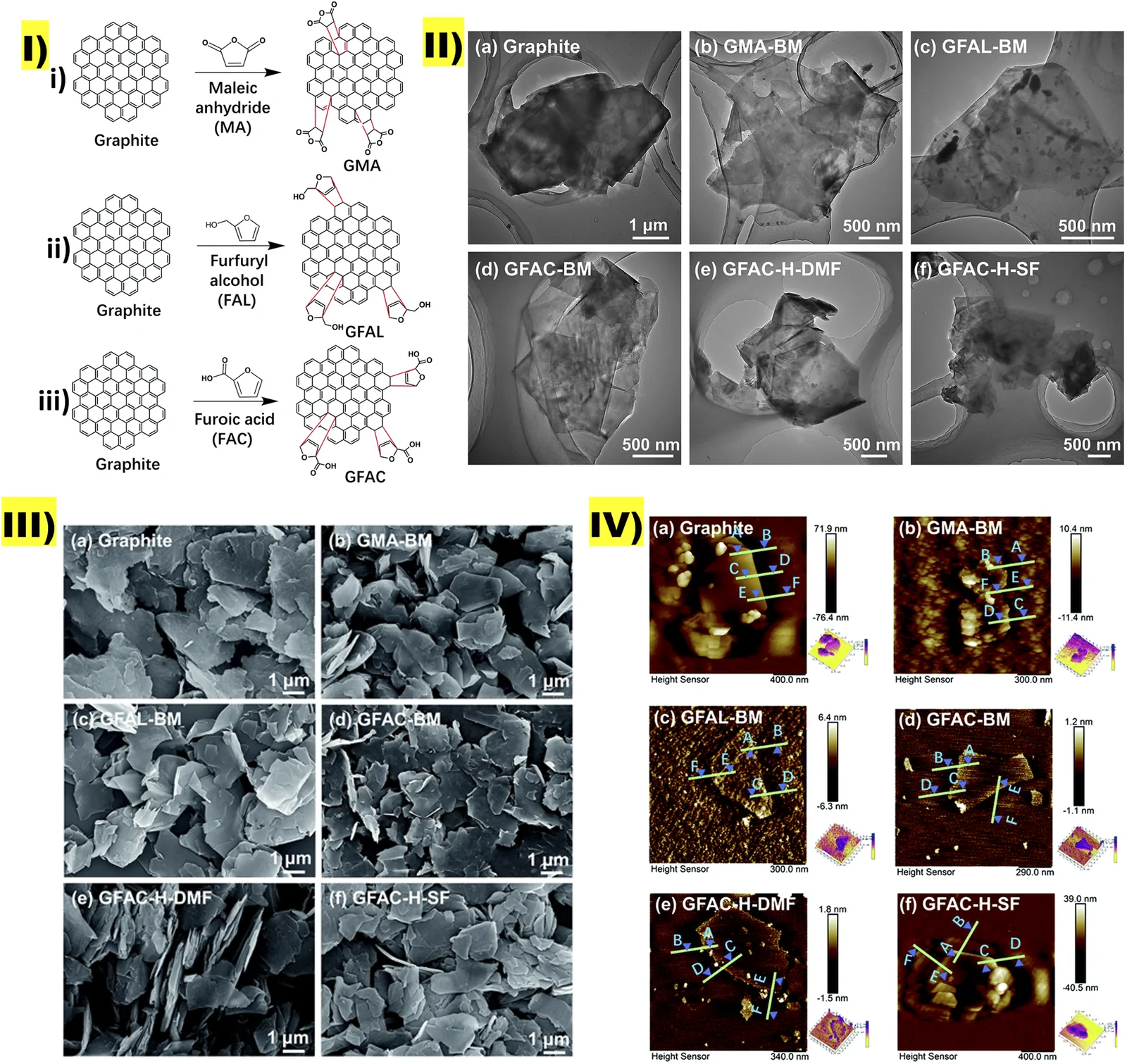
(I) Functionalization of graphene using different furan-based compounds, (II) (a–f) TEM, (III) (a–f) FESEM, and (IV) (a–f) AFM of EFG, reproduced from ref. 82 with permission from The Royal Society of Chemistry,^82^ Copyright 2022.

### 5.4. Thermal stability as an indicator of functionalization

Thermogravimetric analysis (TGA) provides indirect but valuable insight into the nature and extent of functionalization.^113,114^ Edge-bound functional groups typically decompose at moderate temperatures, resulting in minor weight loss events. In contrast, extensive basal-plane oxidation leads to significant mass loss at lower temperatures due to instability of the disrupted lattice.

Mechanochemically derived EFG generally exhibits higher thermal stability (often stable up to ∼400 °C or beyond) compared to graphene oxide, which undergoes major decomposition above ∼200 °C. This difference reflects preservation of the graphitic backbone with limited functional group incorporation.

### 5.5. Structural probing *via* XRD

X-ray diffraction (XRD) provides complementary structural evidence for distinguishing functionalization sites.^115^ Basal-plane functionalization typically increases interlayer spacing, resulting in a noticeable shift of the (002) peak toward lower angles (9–12). In contrast, edge functionalization primarily disrupts stacking order without significantly altering the interlayer spacing, leading instead to peak broadening near ∼26.6°. Additional features, specifically to ball-milled graphene, such as weak reflections in the 42°–44° range and the corresponding multiplicity of spots in selected area electron diffraction patterns, arise from edge-induced distortions or twisting effects, leading to turbostraticity.^116,117^ These observations further support the presence of localized structural perturbations rather than bulk lattice expansion.

### 5.6. Integrated interpretation: edge *vs.* basal functionalization

No single technique is sufficient to conclusively determine the spatial distribution of functional groups in graphene. Instead, a correlative interpretation framework is required. The key correlations for EFG are summarized in Table 3.

**Table 3:** Correlation of experimental observations with inferred functionalization characteristics in graphene

Observation	Inference
Moderate *I*_D_/*I*_G_ + low *I*_D_/*I*_D′_	Edge-dominated defects
Minimal XRD peak shift + peak broadening	Preserved interlayer spacing and edge perturbation
Weak FTIR signals + moderate XPS functional content	Limited functionalization
High thermal stability	Intact graphitic backbone
Raman mapping (edge-localized D-band)	Spatially selective functionalization

Collectively, these signatures indicate that mechanochemical ball milling under optimized conditions promotes edge-selective functionalization while largely preserving the basal sp^2^ carbon network (Table 4). The combined evidence consistently indicates that shear-assisted ball milling enables controlled incorporation of functional groups, predominantly at graphene edges, with minimal disruption to the basal π-conjugated framework. This spatial selectivity is critical for maintaining electronic transport while introducing chemical reactivity, distinguishing mechanochemical routes from conventional oxidation-based approaches.^118^

**Table 4:** Summary table of different ball-milling parameters for graphene production

Sl no	Milling agent	Speed	Time	Post-treatment/temperature	Functional groups	Yield	Ref.
1	Ammonium salts	500 rpm	8 h	Ultrasonication/freeze drying	C–OH and C–NH_2_	2 mg ml^−1^	119
2	High purity graphite treated with KOH		18 h	Cooled at −10 °C	C–OH and C–O–C	—	120
3	Ammonium bicarbonate	500 rpm	12 h	Ultrasonication/freeze drying	C–O and C–NH_2_	—	121
4	Polyetheramine	500 rpm	4 h	Ultrasonication	C–OH, C–O–C, CH_3_, CH, and N–H	—	122
5	Graphite powder & ethanol	500 rpm	12 h	Ultrasonication	Oxygen containing functional groups	—	123
6	Octadecylamine	300 rpm	5 h	Washing & cryogenic drying	C–OH, –NH_2_, C–N, and N–H	—	124
7	Cyclohexanehexone	700 rpm	6 h	Pyrolysis/190 °C	C=O and C–O	—	125
8	Supercritical CO_2_	500 rpm	24 h	Ultrasonication and vacuum filtration	C–OH, C–OOH, and –C–O–C	54%	126
9	Polyvinyl chloride and ethanol	800 rpm	36 h	Vacuum drying	O–H, C–H and C–Cl	—	127
10	Naphthalene	80 rpm	48 h	Pyrolysis/250 °C	No functionalization	46%	128
11	Jaggery	70 rpm	30 h	Ultrasonication	C=O, C–O–C, C–OH, C=C, and C–H	—	129
12	Sucrose	70 rpm	30 h	Pyrolysis/400 °C	C–O, and C=O	93%	130
13	Flour	80 rpm	48 h	Ultrasonication	C–H, C–OH, C–O–C, and C=O	—	131
14	Melamine		30 h	Pyrolysis/550 °C	C=N, C–N, C–NH–C, C–NH_2_, NH, and C–NH–C	—	132
15	Oxalic acid	1250 rpm	20 h	Acid treatment/pyrolysis/600 °C	C=O and COOH	—	133
16	Dry ice	500 rpm	48 h	Acid treatment/freeze drying	C=O, C–O, O=C–OH, C–O, C–OH, C–O–C, and O=C–OH	—	134
17	Sorbitol	80 rpm	48 h	Pyrolysis/400 °C	C–C, C–O–C C=O, –OH, and C–H	15%	135
18	Anthracene	200 rpm	60 min	Pyrolysis/600 °C		—	136
19	Urea	290 rpm	45 h	Washing/vacuum drying	C–C, C–O, C–N, and C=O	—	137
20	Mannitol	500 rpm	8 h	Ultrasonication	–OH, –CH, and –CH_2_	—	138
21	Glucose	100 rpm	30 min	Ultrasonication/dialysis	C–O–C, C=O, C–O–O, and C–C	—	139
22	Starch	80 rpm	48 h	Pyrolysis/400 °C	C–C, –OH, –CH, C–OH, and C–O–C	17%	140

## 6. Tunable edge functionalities and their electrochemical implications

Building upon the established structural and chemical characteristics of EFG, this section focuses on how controlled edge chemistry governs electrochemical performance. The discussion is framed in terms of interfacial processes such as wettability, ion adsorption, charge transport, and redox activity that collectively determine energy storage behavior. In this context, electrochemical performance emerges from a coupled structure–chemistry–property relationship, where defect topology defines the chemical environment, which in turn dictates interfacial electrochemical dynamics.^66^

### 6.1. Edge functional groups and wettability

Edge functionalization introduces polar groups that significantly modify graphene–electrolyte interfacial energetics.^141^ Functional groups such as –OH, –COOH, C=O, and –NH_2_ increase surface polarity and promote hydrogen bonding interactions with electrolyte ions (Fig. 15).^142,143^ As a result, the inherently hydrophobic nature of pristine graphene is mitigated, leading to improved wettability and reduced contact angle.^53^ Enhanced wettability facilitates more effective electrolyte infiltration into electrode structures, thereby increasing the electrochemically accessible surface area and reducing the interfacial resistance. This effect is particularly critical in aqueous electrolyte-based supercapacitor systems, where ion transport is strongly dependent on surface accessibility.^144^ However, the benefit is contingent on spatial selectivity. Functionalization confined to edge regions improves interfacial interaction while preserving conductive pathways, whereas excessive or basal-plane modification can introduce electrode transport limitations.^145^ Thus, wettability enhancement must be achieved without compromising electronic continuity.

**Fig. 15:**
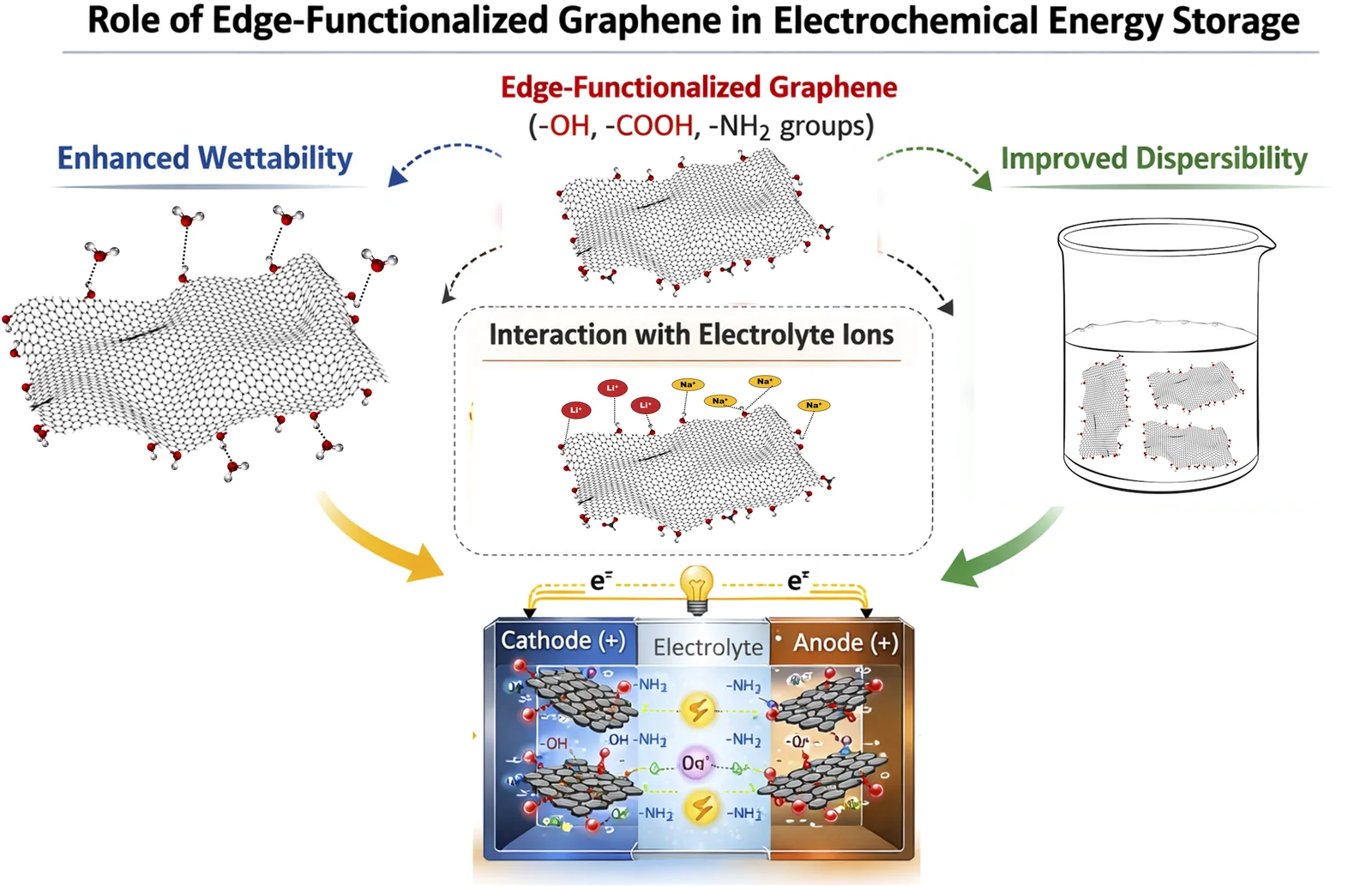
Edge functional groups and their influence on wettability (figure refined using Perplexity AI for illustrative purposes).

### 6.2. Ion adsorption modulation

Ion adsorption behavior in graphene-based electrodes is governed by the local electronic structure and surface chemistry.^146,147^ Edge defects represent energetically favorable adsorption sites relative to the basal plane due to their unsaturated coordination and higher local reactivity.^148^ Functional groups modify adsorption thermodynamics by introducing localized charge redistribution and dipolar interactions. The oxygen-containing groups provide coordination sites for cations, whereas pyridinic nitrogen enhances the electron density and strengthens interaction with Li^+^/Na^+^.^149,150^ The sulfur functionalities can modulate adsorption for larger or multivalent ions.^151^ These chemical modifications alter the electric double-layer structure and can increase the specific capacitance beyond that of pristine graphene.

Nevertheless, excessive functionalization may lead to strong binding interactions that hinder ion mobility or induce irreversible trapping.^152^ Therefore, optimal performance is achieved through controlled functional group density, ensuring a balance between adsorption strength and ion transport kinetics.

### 6.3. Influence on electron transport

Electrical conductivity in graphene is governed by delocalized π-electron transport across sp^2^ domains. Disruption of this network, particularly through basal-plane functionalization, introduces scattering centers and increases charge-transfer resistance.^153^ In contrast, edge-localized functionalization exerts a comparatively limited effect on long-range conductivity, provided that percolating sp^2^ carbon domains remain intact.^145^ This distinction is critical as it allows simultaneous enhancement of chemical activity and retention of electronic transport. The nature of functionalization further influences the conductivity. In-plane oxygen functionalities reduce conductivity *via* sp^2^-to-sp^3^ rehybridization, disturbing the aromatic π-conjugation, whereas graphitic nitrogen can enhance the carrier density and improve the conductivity.^149^ Electrochemical impedance spectroscopy typically reflects these trends. Systems with controlled edge functionalization exhibit low charge-transfer resistance, whereas excessive disorder results in an increased semicircle diameter in Nyquist plots.^154,155^ Thus, maintaining a continuous π-conjugated framework while introducing chemically active edge sites is beneficial for high-performance electrodes.

### 6.4. Surface redox contributions

In addition to electrostatic ion adsorption, edge functional groups can participate in reversible Faradaic reactions, contributing to pseudocapacitance. Oxygen-containing groups such as quinone/hydroquinone pairs undergo redox transitions in aqueous electrolytes, while nitrogen functionalities can introduce additional charge-transfer pathways.^150,156^ These redox-active sites enhance the specific capacitance and energy density compared to purely double-layer systems. In battery chemistries, edge defects can also act as preferential ion storage sites, lowering activation barriers for insertion processes.^58,144,157^ However, the excessive redox-active functionality may compromise structural stability or promote parasitic reactions during cycling. Therefore, controlled incorporation is necessary to achieve reversible and stable electrochemical behavior.

### 6.5. Integrated structure–chemistry–electrochemical framework

The electrochemical performance of ball-milled graphene is governed by the interplay of (a) structural features: edge density, defect topology, and preservation of extended sp^2^ domains, (b) chemical attributes: type, concentration, and distribution of functional groups or heteroatoms, and (c) electrochemical responses: wettability, ion adsorption kinetics, charge-transfer resistance, and pseudocapacitive contributions. Shear-dominant mechanochemical processing provides a distinct advantage by enabling preferential edge functionalization with limited basal-plane disruption, thereby achieving an optimal balance between chemical reactivity and electronic conductivity. This balance is critical for applications requiring both rapid charge transfer and high surface activity, such as high-rate supercapacitors and stable battery electrodes.

Overall, tunable edge functionalization offers a controlled strategy for enhancing electrochemical performance through targeted modification of graphene interfaces.^158^ By regulating functional group chemistry and spatial distribution, it is possible to simultaneously optimize wettability, ion adsorption, charge transport, and redox activity. This approach contrasts with conventional oxidation strategies, where indiscriminate defect generation often compromises conductivity.^159^ Mechanochemical edge engineering thus provides a pathway toward high-performance graphene materials with precisely tailored electrochemical properties.

## 7. Integration into energy storage systems

Mechanochemically engineered graphene thus represents a structurally adaptable and chemically tunable carbon platform for electrochemical energy storage. When translated into device architectures, performance is dictated not only by individual structural features but also by their collective manifestation at the electrode level, where ion transport, charge transfer, and structural stability operate simultaneously. This section focuses on how edge functionality, defect topology, and preservation of conductive domains manifest across different energy storage systems.^160,161^

### 7.1. Supercapacitors

Supercapacitors store energy through electric double-layer capacitance (EDLC) and/or fast surface redox reactions (Fig. 16).^47^ In this context, ball-milled graphene enhances performance primarily through increased electrochemically accessible surface area and improved interfacial dynamics.

**Fig. 16:**
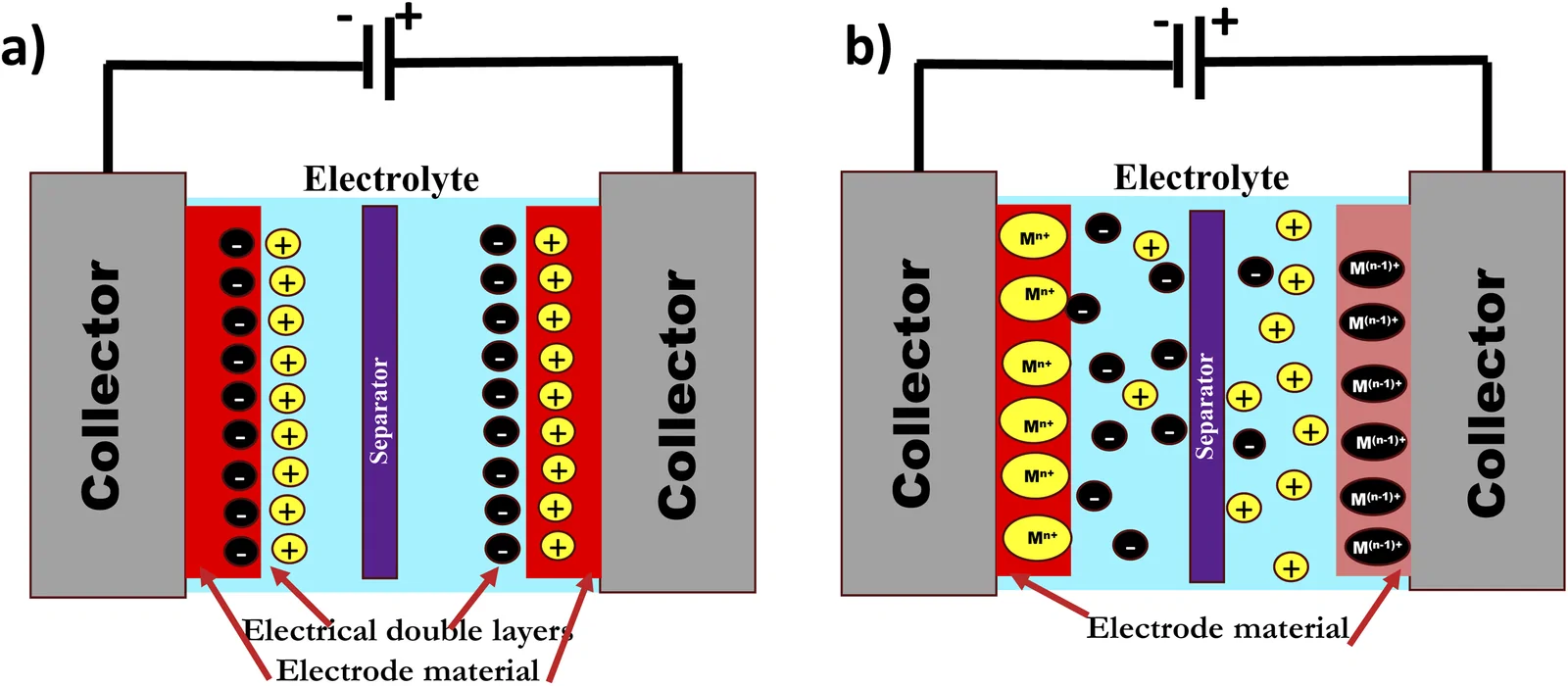
(a) Electric double layer capacitor energy storage mechanism and (b) pseudocapacitor energy storage mechanism.

Exfoliation reduces restacking and increases the ion-accessible surface, while moderate pore formation facilitates electrolyte penetration and shortens ion diffusion pathways.^36,88,91^ At the same time, edge functional groups improve the wettability and reduce the interfacial resistance, particularly in aqueous systems. Nitrogen and oxygen functionalities introduce additional pseudocapacitive contributions *via* surface redox processes.^58^

From a transport perspective, reduced lateral dimensions improve rate capability by minimizing diffusion lengths, provided that sufficient structural continuity is retained. However, excessive defect density or amorphization increases the internal resistance and degrades power performance.^160^ Thus, optimal supercapacitor electrodes require maximized edge accessibility with preserved conductive networks, enabling high capacitance retention at elevated scan rates and long cycling stability.^64,83^ The specific capacitance of selected graphene-based supercapacitor electrodes synthesized *via* ball milling, along with details of the exfoliating agents and post-milling treatments, is summarized in Table 5.

**Table 5:** Comparison of graphene-based electrode materials prepared under different milling conditions and their specific capacitance

SI no.	Electrode material	Milling agents/duration	Post-treatment process	Specific capacitance	Ref.
1	Graphene	Oxalic acid & toluene/20 h	Solvent evaporation and acid treatment	37 F g^−1^	162
2	N-graphyne	CaC_2,_ pyrazine, and ethanol/24 h	Acid treatment and ultrasonication	235 F g^−1^	163
3	Graphene nanosheets	GO, ethanol, and hydrazine/30 min	Ethanol wash and drying	169 F g^−1^	164
4	Microscopic 3D graphene	Coal tar pitch and K_2_CO_3_/15 min	Pyrolysis	182.6 F g^−1^	165
5	Heat-treated edge-carboxylated graphene nanoplatelets	Pristine graphite and dry ice/48 h	Acid treatment and pyrolysis	365.72 F g^−1^	166
6	Graphite carbon material	CaC_2_ and graphite/6 h	Acid treatment	238.4 F g^−1^	167
7	Nanoscale activated carbon from graphite	Surfactant SDBS or SDS/10–30 h	Acid treatment and ultrasonication	1071 F g^−1^	168
8	N-doped graphene	Melamine/48 h	Ultrasonication	75 F g^−1^	169
9	N-doped holey graphene amine	1-Naphthylamine/48 h	Pyrolysis and ultrasonication	12.33 mF cm^−2^	83
10	Carboxymethyl cellulose graphene	Sodium salt of carboxymethyl cellulose/48 h	Pyrolysis and ultrasonication	90 mF cm^−2^	170
11	Carbon sphere/graphene	Table sugar	Pyrolysis and ultrasonication	511 F g^−1^	171

### 7.2. Lithium-ion batteries

In lithium-ion batteries (LIB), charge storage in graphite is traditionally governed by intercalation within ordered graphitic layers. Mechanochemically modified graphene alters this paradigm by introducing additional storage pathways beyond classical intercalation (Fig. 17). Reduced stacking order and expanded interlayer spacing facilitate faster Li^+^ diffusion, particularly under high-rate conditions.^173,174^ Edge sites and defect regions provide additional adsorption and insertion sites to Li ions contributing to surface-controlled storage mechanisms. This leads to enhanced capacity and improved rate performance.^176^

**Fig. 17:**
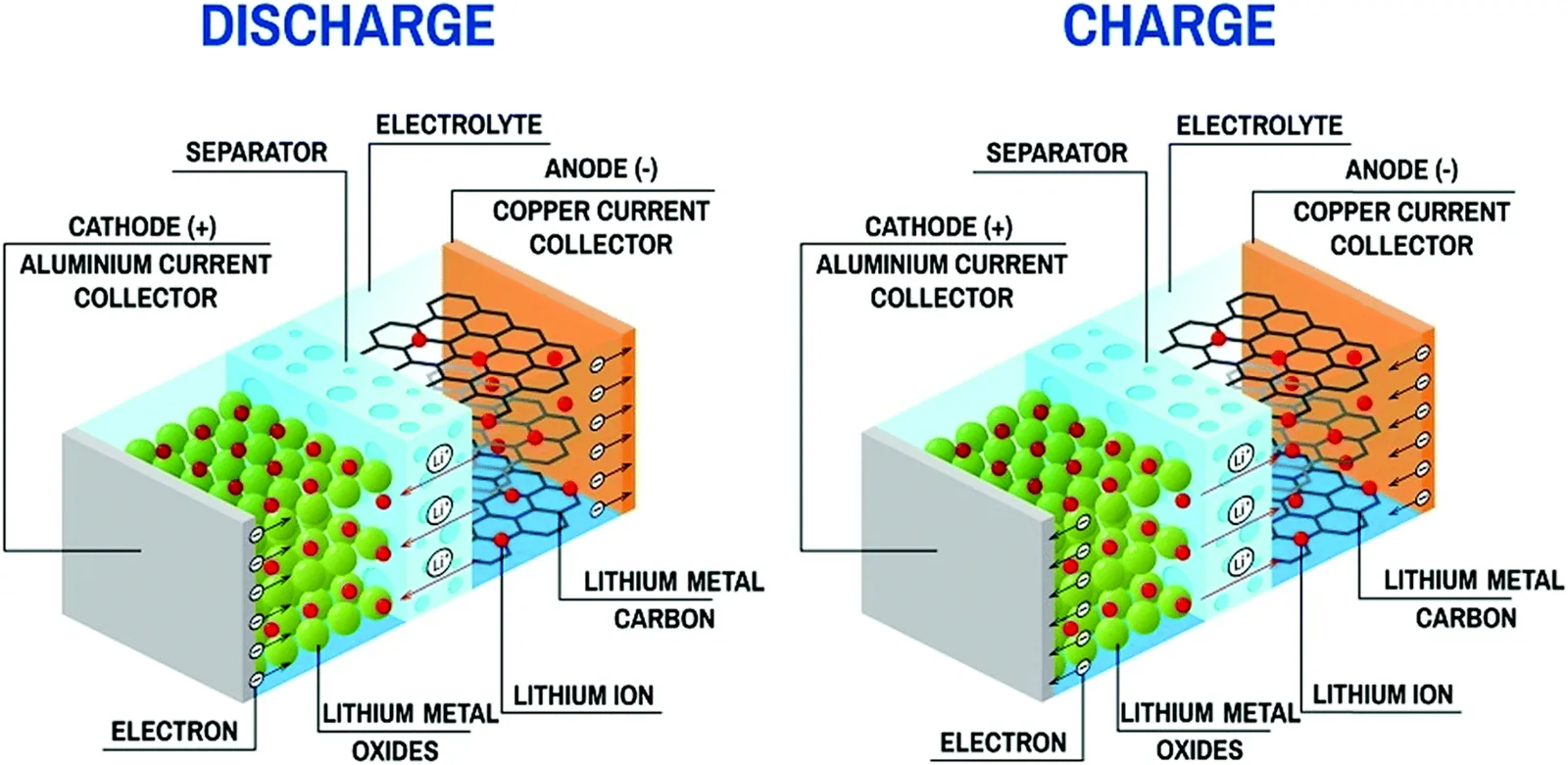
Li-ion battery energy storage mechanism, reproduced from ref. 172 with permission from The Royal Society of Chemistry (Image Credit: https://sivVector/Shutterstock.com),^172^ Copyright 2022.

Moderate functionalization further stabilizes electrode–electrolyte interactions. Oxygen functionalities promote uniform solid electrolyte interphase (SEI) formation, while nitrogen doping can enhance electronic conductivity and modify lithium binding energetics.^45,145,175^ Additionally, defect-engineered graphene serves effectively as a conductive matrix for high-capacity materials (*e.g.*, Si, Sn, *etc*.), buffering volume expansion and maintaining electrical connectivity during cycling.^177^ However, excessive defects increase irreversible capacity loss due to parasitic reactions and unstable SEI formation.^178^ Structural over-fragmentation may also compromise electrode integrity. Therefore, LIB optimization requires controlled defect incorporation that enhances storage kinetics without destabilizing interfacial chemistry or conductivity.

Zhang *et al.* synthesized S-doped graphene using a chemical vapor deposition (CVD) technique followed by ball milling.^179^ Mechanical milling facilitated the incorporation of sulfur atoms into the graphene lattice, thereby enhancing graphene's affinity for sulfur species and improving electrical conductivity, enabling high-performance lithium-ion batteries. Similarly, in another study, graphene foam@CNTs loaded with MoS_2_ nanoparticles improved the contact between the electrode and electrolyte, reduced the Li-ion diffusion distance, and provided internal void spaces to accommodate the volume changes associated with MoS_2_ nanoparticles during cycling.^180^ Liu *et al.* prepared N-doped EFG by the urea-assisted ball-milling method, and it showed 895 mAh g^−1^ specific capacity with a coulombic capacity of 95%.^58^ Sun and his team synthesized nano-Si/graphene nanosheets *via* a discharge-plasma assisted milling, and it delivered a stable reversible capacity of 976 mAh g^−1^ in the fabricated LIB.^181^ This mode of preparation converted graphite into graphene nanosheets with the integration of nano silicon in the *in situ* formed graphene. Therein, the controlled functionalization of graphene helped to maintain its electrical conductivity, enhancing the overall LIB performance.

### 7.3. Sodium-ion batteries

Sodium-ion batteries (SIBs) face intrinsic challenges due to the larger ionic radius and slower diffusion kinetics of Na^+^ compared to Li^+^.^182^ Mechanochemical modification of graphene directly addresses these limitations. Disruption of long-range stacking order and slight expansion of interlayer spacing enable more favorable Na^+^ insertion or adsorption (Fig. 18). Unlike LIBs, sodium storage in graphene is more strongly governed by surface-controlled mechanisms rather than deep intercalation.^183^ Here also, the reduced flake size further shortens ion transport pathways, improving rate performance.

**Fig. 18:**
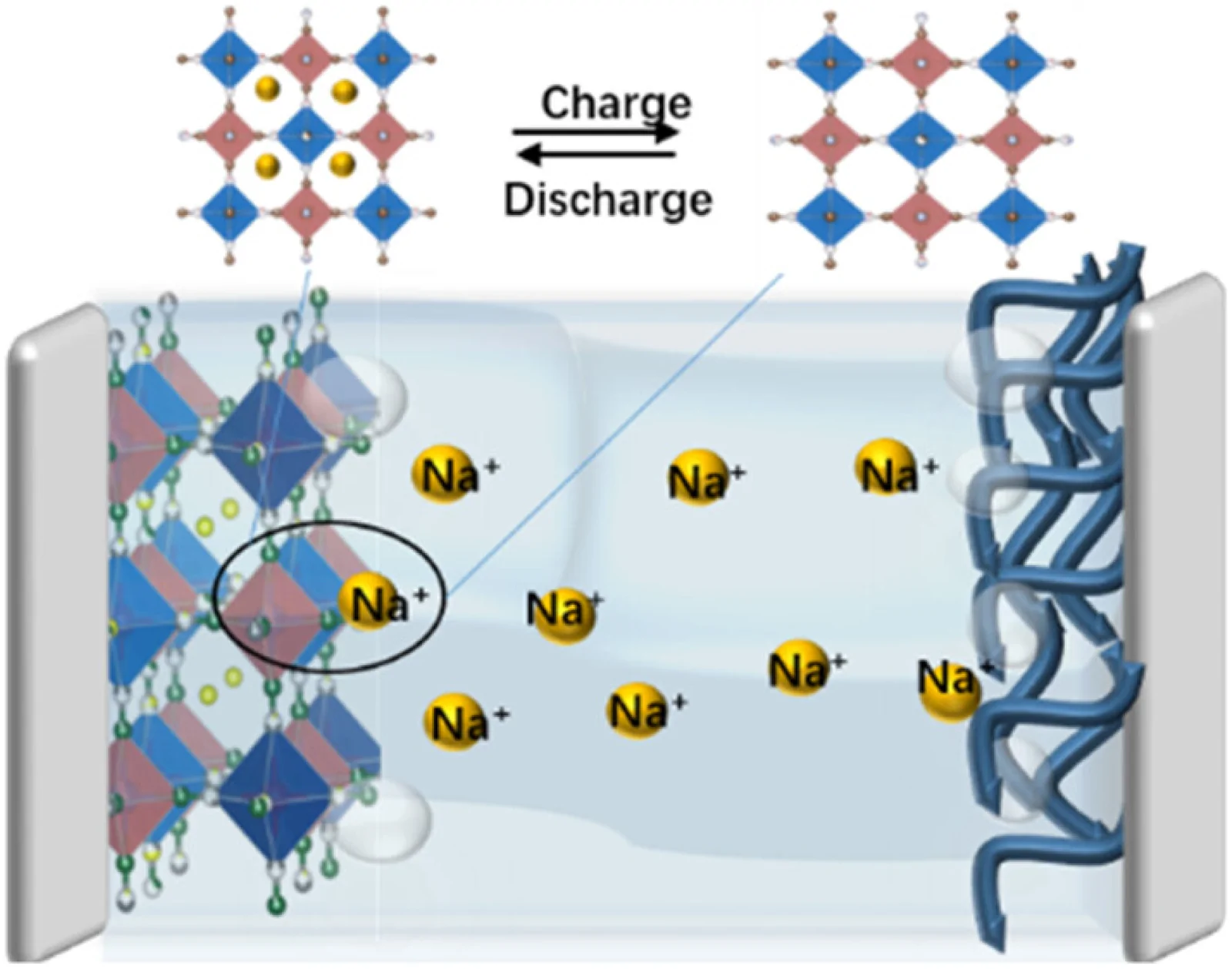
SIB energy storage mechanism, reproduced from ref. 184 with permission from American Chemical Society,^184^ Copyright 2025.

Edge functional groups and heteroatom doping enhance sodiophilicity by modifying the local charge distribution and adsorption energetics.^185,186^ These features promote reversible Na^+^ storage through combined adsorption and pseudocapacitive processes. As with lithium systems, excessive amorphization reduces conductivity and structural robustness.^187^ Optimal SIB electrodes therefore require edge-disordered but electronically continuous graphene frameworks with moderate functional group density, enabling efficient ion interaction without compromising stability.

Zhang *et al.* developed holey graphene nanoplatelets enriched with oxygen-containing functional groups by annealing lithiated graphite from waste LIB under a mixed H_2_O/Ar atmosphere, followed by ball milling in Ar. Used as a SIB anode, this material delivered a high specific capacity of 416.1 mAh g^−1^ at 0.03 A g^−1^.^188^ Dong *et al.* regulated the defect density of graphene through a ball-milling approach to enhance Na^+^ storage performance.^189^ The defect-engineered graphene exhibited a high specific capacity of 507 mAh g^−1^ at a current density of 50 mA g^−1^, along with excellent rate capability. Furthermore, the device delivered an ultrahigh energy density of 45 Wh kg^−1^ even at a high-power density of 14 205 W kg^−1^. Closed micropores within the graphene sheets enabled sodium clustering, while surface defects and functional sites contributed to the capacitive adsorption of sodium ions during the charge–discharge process.^190^

### 7.4. Zinc-ion energy storage systems

Aqueous zinc-ion batteries and hybrid zinc-ion capacitors offer advantages in safety, cost, and environmental compatibility.^191,192^ However, the divalent nature and high charge density of Zn^2+^ impose stringent requirements on electrode materials. Ball-milled graphene provides a suitable platform by combining high surface accessibility with tunable surface chemistry. Edge sites facilitate Zn^2+^ adsorption, while improved wettability enhances electrolyte penetration and reduces interfacial resistance. Structural porosity further supports rapid ion transport (Fig. 19).

**Fig. 19:**
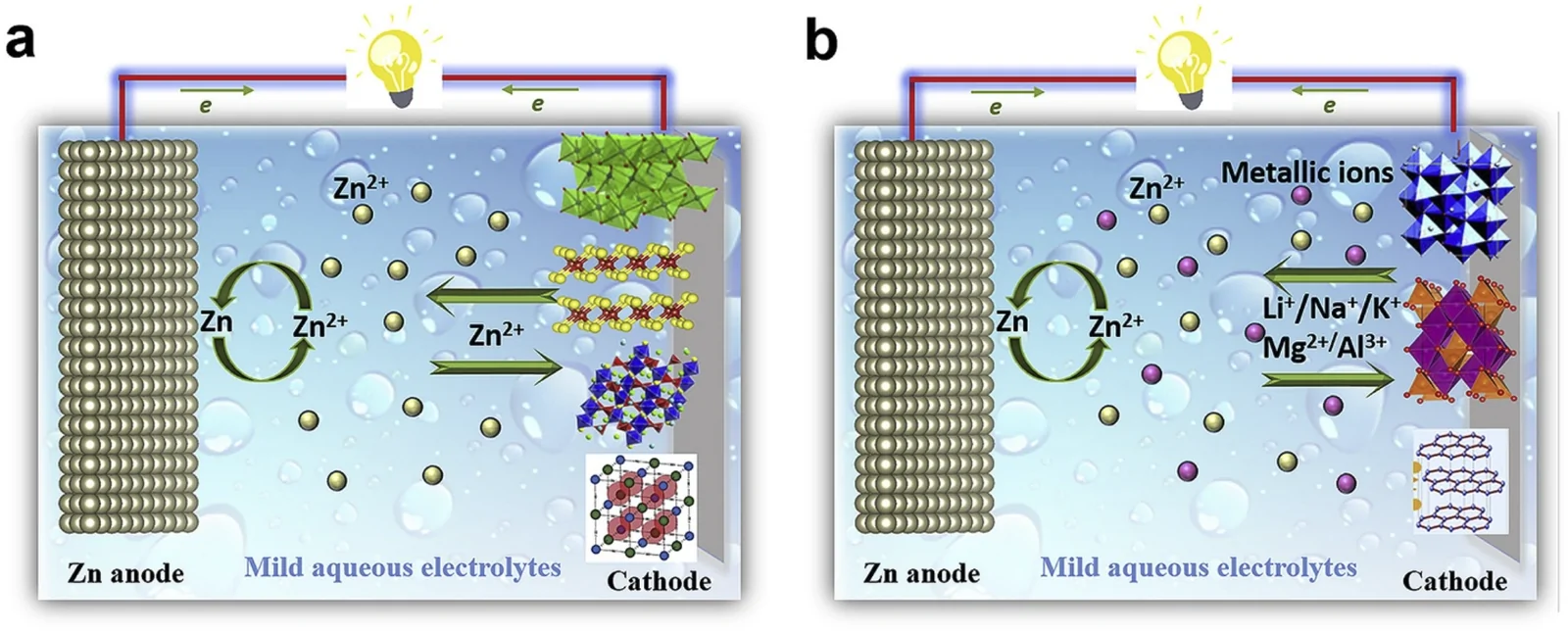
Energy storage mechanisms of (a) aqueous zinc ion batteries and (b) aqueous zinc hybrid batteries, reproduced from ref. 193 with permission from Elsevier B.V.,^193^ Copyright 2019.

Chemical functionalization plays a critical role in regulating Zn^2+^ interactions. Edge heteroatoms modulate the local charge distribution and stabilize adsorption processes, improving reaction reversibility.^194^ Additionally, when used as a conductive scaffold, defect-engineered graphene promotes uniform current distribution, mitigating dendritic growth and improving cycling stability.^195,196^ Nevertheless, highly defective structures can accelerate side reactions in aqueous environments. Therefore, zinc-ion systems particularly require balanced functionalization that enhances ion interaction while maintaining chemical stability under aqueous conditions.^197^

Etesami *et al.* synthesized N-doped EFG with trimetallic NiFeCo *via* ball-milling carbon nanotubes, metal salts, and melamine. Used as a zinc–air battery electrode, the composite demonstrated stable cycling at 10 mA cm^−2^, with the voltage gap increasing by only 0.32 V after 350 cycles.^198^ Zhang *et al.* synthesized oxygen-doped porous graphitized carbon by ball-milling tannic acid with Fe-salt catalysts. The resulting structure provided interconnected transport pathways and abundant electroactive sites, enhancing Zn^2+^ adsorption kinetics. This electrode achieved a specific capacity of 216 mAh g^−1^ at 0.5 A g^−1^, highlighting ball milling as a versatile, cost-effective route for high-performance zinc-ion supercapacitors.^199^

### 7.5. Hybrid supercapatteries

Hybrid supercapatteries integrate battery-type and capacitive electrodes to simultaneously achieve high energy and high power density (Fig. 20).^200,201^ In such systems, ball-milled graphene plays a dual role as both an active material and a conductive scaffold. Few-layer graphene structures provide rapid electron transport and mechanical flexibility, while edge functional groups enhance interfacial coupling with redox-active materials such as metal oxides, sulfides, or conducting polymers.^202,203^ This improves the charge-transfer efficiency and enables synergistic operation between faradaic and capacitive processes. Defect-induced pseudocapacitance further contributes to charge storage, bridging the performance gap between conventional supercapacitors and batteries. At the same time, preserved graphitic domains maintain high-rate capability.^160^ However, excessive functionalization may induce structural instability under repeated redox cycling. Therefore, hybrid systems benefit from precise tuning of edge density and functional group concentration, ensuring durability alongside enhanced performance.

**Fig. 20:**
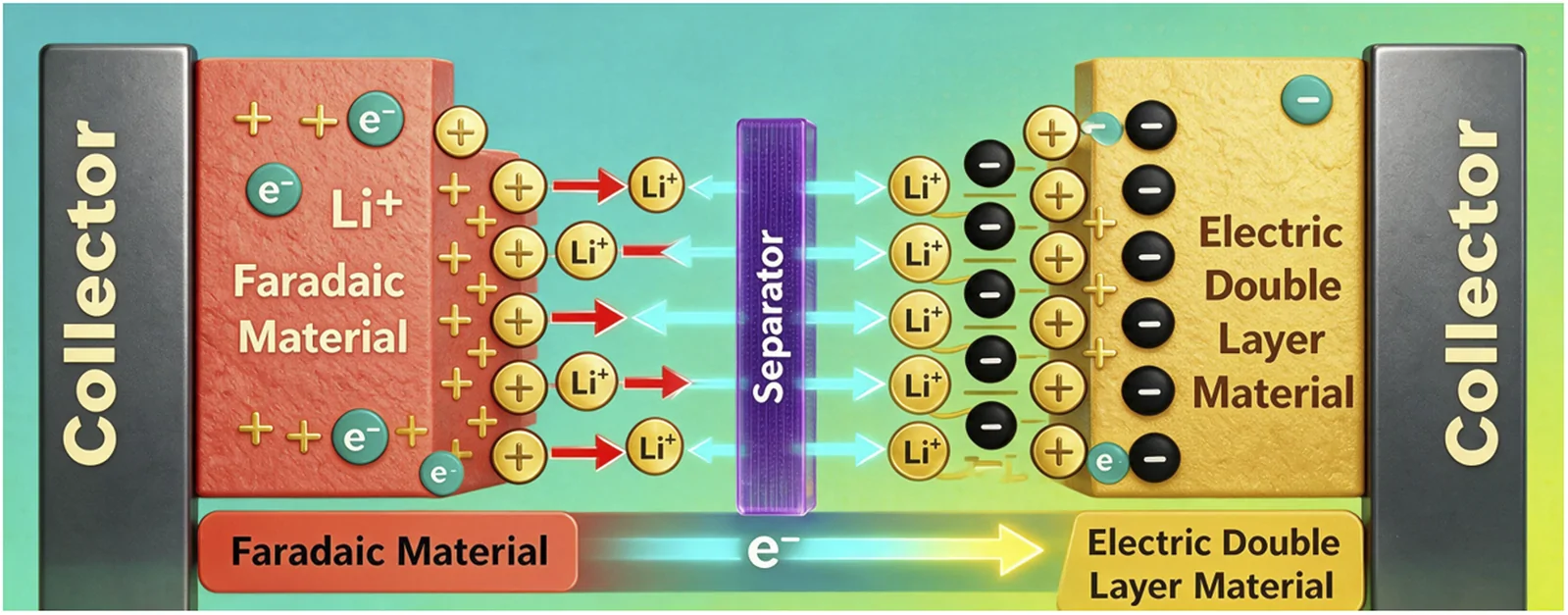
Hybrid supercapattery energy storage mechanism (figure refined using Perplexity AI for illustrative purposes).

Additionally, a few reports on ball-milled graphene in individual capacitive and battery-type electrodes are discussed here to provide further insights into its potential role in hybrid supercapatteries. Jung *et al.* utilized ball-milling and water oxidation to produce smaller reduced graphene oxide (rGO) sheets for self-supporting, high-performance supercapacitor electrodes. This method enhance ion transport and increase the edge plane density, achieving a capacitance of 117.9 F g^−1^.^204^ Shi *et al.* synthesized porous Si/carbon composites by ball-milling silicon with graphite and NaCl. The resulting pyrolytic carbon coating and NaCl-derived pores stabilized the solid electrolyte interphase, enhanced conductivity, and shortened Li^+^ diffusion pathways while buffering volume expansion.^205^ Zhang *et al.* ball-milled GO with acetone to prevent agglomeration and induce exfoliation while removing unstable oxygen groups. The resulting graphene featured enriched edge functionalities and improved conductivity, delivering a capacitance of 212 F g^−1^ through combined double-layer and pseudocapacitive storage.^206^ Dong *et al.* synthesized a defect-enriched graphene block (DGB) *via* 8 hours of ball milling, inducing “self-doping” defects that enhance ion storage through electric double-layer behavior. This approach produces EFG, boosting electrochemical performance by increasing active edge sites and improving conductivity.^207^

Across all energy storage systems, the performance of ball-milled graphene is governed by a unified set of interdependent parameters, as described in Fig. 21.

**Fig. 21:**
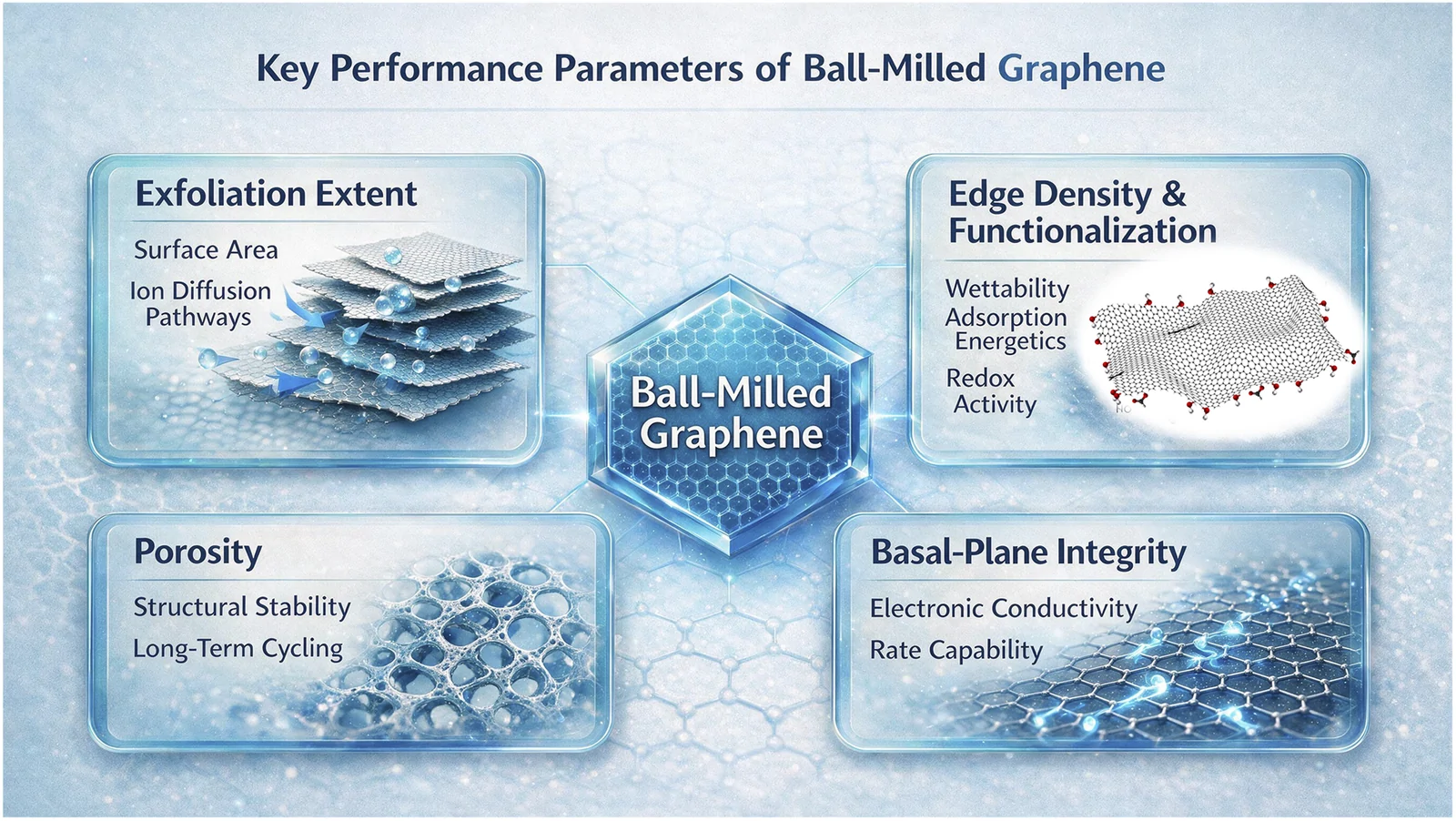
Dependence of structural parameters on the electrochemical performance of ball-milled graphene (figure generated using Perplexity AI for illustrative purposes).

Mechanochemical processing uniquely enables simultaneous tuning of these parameters within a single synthesis platform. The central challenge is not maximizing any single attribute, but achieving an optimal balance tailored to the dominant charge storage mechanism of the target system.^142–144^ Integration of ball-milled graphene into energy storage devices highlights the importance of coupling structural design with electrochemical functionality. By enabling controlled edge functionalization while preserving conductive pathways, mechanochemical processing provides a scalable route to high-performance electrode materials. System-specific optimization of defect topology and surface chemistry is essential to fully realize the potential of graphene across diverse energy storage technologies. Based on the available literature, milling-agent-assisted ball mill exfoliation of graphite remains largely underexplored across diverse energy storage systems. Most existing studies are predominantly confined to supercapacitor applications, thereby revealing substantial scope for expanding this approach into broader energy storage technologies and uncovering new research opportunities.

## 8. Scalability, process economics, and industrial viability

Translating ball-milled graphene from laboratory-scale demonstrations to industrial deployment necessitates a rigorously quantified assessment of process energetics, production efficiency, economic feasibility, and environmental compatibility. Unlike conventional chemical exfoliation routes, shear-dominated mechanochemical processing operates within a solid-state framework where structural evolution is governed by controlled mechanical energy input rather than chemical transformation pathways.^208,209^ This distinction fundamentally alters both the cost structure and scalability considerations. Accordingly, this section evaluates the industrial relevance of the process through key parameters including energy intensity, yield quality, economic distribution, and system integration.

### 8.1. Energy consumption of milling

In shear-governed milling regimes, energy transfer occurs predominantly through sustained frictional and rolling interactions, enabling progressive layer delamination and edge activation.^49^ A meaningful evaluation of process efficiency requires normalization of energy consumption in terms of specific energy input (kWh kg^−1^), directly correlating power utilization with the quantity of structurally functional graphene produced. This metric is strongly influenced by operational variables such as milling duration, rotational dynamics, ball-to-powder ratio, media characteristics, and reactor loading conditions.^36,39,74^ From a process optimization standpoint, the objective is not maximal exfoliation, but attainment of a defined structural state characterized by targeted layer thickness, edge density, and defect distribution. Beyond this threshold, continued milling contributes disproportionately to energy consumption while offering negligible improvement, or even degradation, in electrochemical functionality. Integration of intermittent structural diagnostics (*e.g.*, Raman spectroscopy, surface area analysis, *etc*.) can therefore serve as a feedback mechanism to terminate milling at energetically optimal points. At an industrial scale, where electrical demand constitutes a major fraction of operating cost, minimizing energy intensity per unit of functional product becomes a primary determinant of process viability.

### 8.2. Yield considerations

Yield assessment in mechanochemical graphene production must extend beyond simple mass recovery to include structural and functional qualification of the product. Accordingly, two complementary metrics are defined: (i) mass yield, representing material retention throughout processing, and (ii) functional yield, denoting the fraction of output meeting predefined structural and electrochemical criteria.^210^ Shear-dominated milling inherently favors high material retention due to the absence of solvent-phase processing and associated separation losses. More importantly, the gradual nature of mechanical exfoliation preserves graphitic continuity while selectively generating edge defects, thereby maximizing the proportion of the electrochemically active material. Functional yield, however, is governed by the reproducibility of key structural attributes, including layer distribution, defect topology, and surface chemistry. Variability in these parameters directly translates into fluctuations in device performance. Consequently, process control, rather than increased mechanical intensity, emerges as the central lever for achieving consistent, application-grade graphene.

### 8.3. Cost analysis

The economic framework of shear-driven ball milling is fundamentally distinct from that of solution-based graphene synthesis, as it eliminates the need for extensive chemical handling, solvent recovery, and effluent treatment infrastructure. As a result, the cost structure is more heavily weighed toward mechanical and energy inputs rather than consumable reagents.^164^ Primary cost components include raw graphite procurement, electrical energy consumption, capital investment in milling systems, and recurring operational expenses such as maintenance, labor, and media replacement. Among these, electrical energy represents the most dynamic variable, as it directly governs both exfoliation kinetics and defect engineering efficiency.^32,208,211,212^ Capital expenditure is influenced by mill design, capacity, and process monitoring capabilities, but remains comparatively streamlined due to the absence of corrosion-resistant chemical processing units. Operational costs are closely linked to media wear, which not only affects the replacement frequency but also introduces potential contamination pathways that must be managed through material selection and process design.

A robust techno-economic evaluation must integrate these factors into a unified model incorporating the energy intensity, yield efficiency, equipment depreciation, and quality rejection rates. Importantly, economic feasibility is application-dependent: graphene used as a conductive additive tolerates higher defect levels and lower production costs, whereas electrochemically active materials demand tighter structural control, increasing production complexity and cost thresholds.^35,211^ Post-treatment process if required is the other cost-increasing factor.

### 8.4. Green chemistry and sustainability

Mechanochemical exfoliation offers intrinsic environmental advantages by operating predominantly in the solid state and minimizing reliance on hazardous reagents.^213^ The near-elimination of liquid-phase processing significantly reduces wastewater generation and simplifies downstream purification requirements.^32,210^ However, the overall environmental footprint is not negligible and is largely dictated by the source and efficiency of electrical energy consumption. Integration of renewable energy inputs can substantially lower lifecycle emissions, whereas reliance on carbon-intensive power sources offsets the environmental benefits of solvent-free processing.

Material sustainability is further influenced by the durability of milling components and the extent of particulate contamination. Selection of wear-resistant media and optimization of operating conditions extend equipment lifespan while preserving product purity. Additionally, acoustic emissions during milling necessitate mitigation through soundproof enclosures and vibration-damping systems to ensure occupational safety and compliance with industrial noise standards. A comprehensive sustainability assessment must therefore encompass the full lifecycle, from graphite sourcing and mechanical processing to device integration and end-of-life considerations; in addition, the post-treatment process also needs to stick on pollutant-free methodologies or low emission platforms. Notably, the extended operational lifetime of energy storage devices enabled by structurally optimized graphene can compensate for production-related impacts, reinforcing the importance of performance-driven sustainability.

### 8.5. Continuous *vs.* batch processing and monitoring

Batch milling offers considerable flexibility for controlling processing conditions and tailoring the graphene structure, facilitating it as a facile approach for laboratory-scale studies and customized material development. Its discrete mode of operation allows systematic variation of milling parameters and facilitates detailed investigation of structure–property relationships. For large-scale production, semi-continuous and continuous processing approaches may provide advantages in terms of workflow integration and operational efficiency. However, their application to graphene production is not straightforward. Effective exfoliation and edge functionalization often require prolonged milling durations, frequently extending over several tens of hours. Reproducing the same shear-dominant mechanical environment and ensuring sufficient material residence time under continuous operation therefore remain important considerations during process design and scale-up.

Integration of milling with downstream manufacturing steps, such as electrode fabrication, composite preparation, or powder-processing operations, can simplify production workflows and reduce intermediate handling requirements. Such process integration may contribute to improved manufacturing efficiency and product consistency. Process monitoring is equally important for maintaining reproducibility. Parameters such as milling torque, reactor temperature, vibration behaviour, and material flow characteristics can provide valuable information regarding process stability. The use of real-time monitoring tools enables early identification of operational deviations and supports consistent control over product quality. Consequently, effective monitoring and process control will be essential for translating laboratory-scale mechanochemical graphene production into reliable manufacturing practice.

### 8.6. Overall industrial evaluation

Shear-dominant mechanochemical exfoliation represents a technically viable and industrially adaptable route for producing EFG with controlled structural attributes. Its strength lies in the ability to simultaneously modulate exfoliation, defect generation, and surface chemistry within a single processing step, without reliance on complex chemical processes. Industrial feasibility, however, is contingent upon achieving a precise balance between energy efficiency, functional yield, and throughput. The process must consistently deliver graphene with application-specific structural characteristics while maintaining cost competitiveness.

Ultimately, large-scale adoption will depend not only on laboratory-scale performance metrics but also on validated techno-economic models, process reliability, and lifecycle sustainability. When these factors are systematically aligned, mechanochemical exfoliation offers a credible pathway toward scalable production of graphene tailored for advanced electrochemical energy storage applications.

## 9. Challenges and future research directions

Ball mill-derived EFG has evolved from an exploratory mechanochemical concept into a controllable and increasingly reproducible materials platform. With consistent edge chemistry and mature characterization methodologies now available, the field is gradually transitioning from proof-of-concept studies toward precision engineering and application-oriented optimization. Nevertheless, several challenges remain before the full potential of this approach can be realized. These include controlling defect generation without inducing excessive amorphization, minimizing contamination arising from milling media, narrowing the broad distributions in flake size and layer thickness commonly observed after milling, improving reproducibility across different milling systems, reducing energy consumption associated with prolonged processing, and addressing scale-up constraints while maintaining product quality. Future research should therefore focus not only on enhancing material performance but also on improving process robustness, efficiency, and industrial relevance.

A key priority moving forward is the development of standardized and interoperable frameworks for evaluating structural evolution and defect chemistry in ball-milled graphene. While Raman spectroscopy remains one of the most widely used tools for assessing disorder and exfoliation, meaningful interpretation often requires correlation with complementary techniques such as X-ray photoelectron spectroscopy (XPS), Fourier-transform infrared (FTIR) spectroscopy, X-ray diffraction (XRD), transmission electron microscopy (TEM), and electrical conductivity measurements. Establishing harmonized reporting practices for parameters such as defect density, functional-group content, layer number distribution, lateral flake dimensions, and degree of exfoliation would greatly improve comparability among studies and facilitate the development of predictive structure–property relationships. Such standardization is particularly important, given the wide variation in milling conditions, reactor configurations, and characterization protocols currently employed across the literature.

From a process-engineering perspective, improving reproducibility and process efficiency remains a significant challenge. Although moderate-speed, shear-dominant milling generally offers improved controllability compared with highly energetic impact-dominated regimes, prolonged milling durations can increase operational costs and energy demand. In addition, wear of milling jars and media may introduce metallic or ceramic contaminants, especially during extended processing, potentially influencing both material properties and electrochemical performance. Future studies should therefore focus on optimizing reactor configurations, milling media, and operating conditions to minimize contamination while maximizing the exfoliation efficiency. Advanced modelling approaches, including discrete element method (DEM) simulations, together with torque, temperature, and power monitoring, may provide valuable insights into stress distribution, energy transfer, and structural evolution, thereby reducing reliance on empirical optimization.

Another important direction involves adapting ball-milling processes to larger-scale manufacturing. While batch processing remains highly valuable for parameter optimization and specialized material development, industrial implementation will require improved throughput and process consistency. Continuous and semi-continuous milling configurations offer attractive possibilities in this regard. However, graphite exfoliation often requires extended processing times, and maintaining the shear-dominated conditions responsible for controlled edge formation during continuous operation remains a non-trivial challenge. Future work should therefore focus on reactor designs capable of balancing productivity with structural control, ensuring that increased throughput does not compromise exfoliation quality or defect management.

At the materials-design level, future research is expected to move beyond maximizing exfoliation or reducing defect density toward establishing application-specific structure–property maps. The objective should be identifying the optimal balance between exfoliation, defect generation, edge functionalization, and preservation of graphitic order for different electrochemical technologies. Such relationships would enable predictive material design rather than empirical optimization, allowing graphene structures to be tailored more precisely for supercapacitors, lithium-ion batteries, sodium-ion batteries, zinc-ion systems, hybrid energy-storage devices, and emerging electrochemical technologies. A deeper understanding of how different edge functionalities, heteroatom dopants, and defect topologies influence electrochemical processes will further strengthen this design-driven approach.

Sustainability considerations will also become increasingly important as the field progresses toward practical deployment. Although mechanochemical exfoliation is inherently more environmentally benign than many oxidation–reduction routes, opportunities remain to reduce energy consumption, improve the milling efficiency, extend the media lifetime, and integrate renewable energy sources into production workflows. Comprehensive techno-economic and life-cycle assessments will be essential for identifying realistic pathways toward commercial implementation and for benchmarking mechanochemical routes against competing graphene-production technologies.

Overall, the future development of ball mill-derived EFG will depend on the successful integration of precise structural control, standardized characterization, reproducible processing, and scalable manufacturing strategies. Continued efforts to address contamination, size distribution, amorphization, energy efficiency, and scale-up challenges will be critical for translating laboratory-scale advances into industrially relevant technologies. By addressing these interconnected challenges, ball milling can evolve from a versatile synthesis technique into a predictable, sustainable, and economically viable manufacturing platform for advanced graphene materials tailored to next-generation energy-storage applications.

## 10. Summary and conclusions

Shear-driven ball milling has emerged as an effective mechanochemical approach for producing edge-functionalized graphene (EFG), offering a practical combination of graphite exfoliation, controlled defect generation, and surface functionalization. In contrast to oxidation-based routes that often introduce extensive basal-plane damage, shear-dominant milling promotes gradual layer delamination while preferentially generating reactive edge sites. This enables the production of high-quality graphene with controlled functionality while largely preserving the sp^2^-carbon π-conjugated framework that is essential for efficient charge transport. The structural evolution of graphite during ball milling is strongly influenced by the interplay of milling conditions, atmosphere, and milling-agent chemistry. By carefully tuning these parameters, it is possible to regulate exfoliation, edge formation, pore development, heteroatom incorporation, and surface functionality. Characterization techniques such as Raman spectroscopy, XPS, FTIR, XRD, and electron microscopy have provided valuable insights into these transformations and have enabled differentiation between edge-dominated functionalization and extensive basal-plane modification. The electrochemical performance of ball-milled graphene is closely linked to this controlled edge engineering. Edge functionalities improve electrolyte wettability, facilitate ion access, and can contribute additional redox-active sites, while the preserved graphitic domains maintain electrical conductivity. As a result, EFG prepared through ball milling has shown considerable promise for supercapacitors, hybrid supercapatteries, lithium-ion batteries, sodium-ion batteries, zinc-ion systems, and other emerging energy-storage technologies. In addition to its electrochemical advantages, shear-driven ball milling offers attractive features from a manufacturing perspective, including process simplicity, scalability, reduced chemical consumption, and compatibility with more sustainable production practices. Although further work is required to establish quantitative process–structure–property relationships and standardized characterization protocols, significant progress has already been made toward understanding the mechanistic foundations of this approach.

Overall, shear-driven ball milling has evolved beyond a simple graphite exfoliation technique into a versatile platform for engineering graphene structure and functionality. Its ability to generate controlled edge chemistry while preserving the electronic integrity of the basal plane provides a distinctive advantage for energy-storage applications. Continued advances in process optimization, mechanistic understanding, and application-oriented material design are expected to further expand the potential of ball-milled EFG and support its translation from laboratory research into practical technologies.

## Author contributions

Sumisha S.: manuscript – original draft preparation. Binitha N. Narayanan: supervision, validation, manuscript – reviewing and editing.

## Conflicts of interest

The authors declare that we have no conflicts of interest in this work. We declare that we do not have any commercial or associative interest that represents a conflict of interest in connection with the work submitted.

## Data Availability

No primary research results, software or code have been included and no new data were generated or analysed as part of this review.
